# Identifying the Genetic Basis of Mineral Elements in Rice Grain Using Genome-Wide Association Mapping

**DOI:** 10.3390/genes13122330

**Published:** 2022-12-10

**Authors:** A. S. M. Faridul Islam, Wardah Mustahsan, Rodante Tabien, Joseph M. Awika, Endang M. Septiningsih, Michael J. Thomson

**Affiliations:** 1 Department of Soil and Crop Sciences, Texas A&M University, College Station, TX 77843, USA; 2 Department of Agronomy, Kansas State University, Manhattan, KS 66506, USA; 3 Texas A&M AgriLife Beaumont Research Center, Beaumont, TX 77713, USA

**Keywords:** GWAS, SNP, rice grain, minerals

## Abstract

Mineral malnutrition is a major problem in many rice-consuming countries. It is essential to know the genetic mechanisms of accumulation of mineral elements in the rice grain to provide future solutions for this issue. This study was conducted to identify the genetic basis of six mineral elements (Cu, Fe, K, Mg, Mn, and Zn) by using three models for single-locus and six models for multi-locus analysis of a genome-wide association study (GWAS) using 174 diverse rice accessions and 6565 SNP markers. To declare a SNP as significant, −log10(P) ≥ 3.0 and 15% FDR significance cut-off values were used for single-locus models, while LOD ≥ 3.0 was used for multi-locus models. Using these criteria, 147 SNPs were detected by one or two GWAS methods at −log10(P) ≥ 3.0, 48 of which met the 15% FDR significance cut-off value. Single-locus models outperformed multi-locus models before applying multi-test correction, but once applied, multi-locus models performed better. While 14 (~29%) of the identified quantitative trait loci (QTLs) after multiple test correction co-located with previously reported genes/QTLs and marker associations, another 34 trait-associated SNPs were novel. After mining genes within 250 kb of the 48 significant SNP loci, in silico and gene enrichment analyses were conducted to predict their potential functions. These shortlisted genes with their functions could guide future experimental validation, helping us to understand the complex molecular mechanisms controlling rice grain mineral elements.

## 1. Introduction

Being a staple food for half of the world’s population, the nutritional quality of rice can have a large impact on human nutrition. The lack of nutritional quality mainly affects countries where rice is eaten primarily as a staple food. Mineral malnutrition is one of the more serious problems for rice-eating societies, especially in Asian countries [1]. More than 60% and 30% of the world’s population have iron (Fe) and zinc [2] deficiency, respectively, because of low mineral content availability in their staple foods, including rice [3,4]. Additionally, other minerals are necessary for human health. For instance, Mg is needed to generate energy from ATP, and it involves in neuromuscular function and cardiac cycle [5,6]. Lack of K causes hypokalemia, paralysis, and cardiac disorders [6]. Cu is involved in bone formation and red blood cell production. It has antioxidant features, controlling free radicals in human body [6]. Mn deficiency leads to faulty bone formation, glucose intolerance, alopecia, and dermatitis [6]. To solve these mineral malnutrition issues, dietary diversification, supplementation, fortification, and biofortification have been practiced so far [7]. Biofortification is the strategy of improving the nutrient content in staple crops through agronomic practices, conventional plant breeding or transgenic approaches [7,8]. Generally, plant breeding or transgenic approaches are more convenient in terms of long-run cost-effectiveness and easy access to the neediest people by developing new varieties with high concentrations of minerals [1,9]. Towards that end, genetic mechanisms controlling the accumulation of the mineral elements in rice grains need to be studied thoroughly.

A number of linkage mapping studies have investigated QTLs controlling mineral accumulation in rice grain. Over 200 QTLs have been identified for micro (Fe, Zn, Mn, Cu) and macro (Ca, Mg, P and K) nutrients elements [10]. However, biparental QTL studies have several limitations. Biparental mapping populations only have the ability to evaluate two alleles per locus, while multiple alleles per locus may exist in diverse natural populations. Moreover, due to the limited resolving power from a low number of recombination events, the identified QTLs are often found in large genomic regions, making it very difficult to pinpoint the causal genes without expensive and time-consuming fine-mapping and map-based cloning efforts [11,12].

Association mapping based on linkage disequilibrium (LD), as an alternative approach to linkage mapping, is a powerful method for dissecting the genetic basis of plant traits [13]. This approach has several advantages, including: (1) it permits the use of natural populations instead of cross-fertilized mapping populations that take time and money to develop; (2) it can detect more than two alleles per locus and (3) it enables a high resolution of mapping. Though it is a promising technique, there are still some drawbacks; for example, large population sizes are needed to provide statistical power to detect rare alleles; likewise, many markers are required to provide high resolution, and population structure between accessions needs to be controlled [13]. Initially, genome-wide association studies (GWAS) were applied in human genetics and then successfully introduced in various plant species [13]. This technique has also been used widely in rice, starting in 2010 by Huang et al. [14] using GWAS to detect QTLs for 14 agronomic traits.

To conduct GWAS, several statistical models have been widely used, including the general linear model (GLM) and the mixed linear model (MLM) [15]. The MLM is the most popular due to its ability to account for population structure and family relatedness. The Efficient Mixed-Model Association eXpedited (EMMAX), Population Parameters Previously Determined (P3D), and Genome-wide Efficient Mixed Model Association (GEMMA) have been developed based on MLM, helping to reduce the computational time for analysis [16]. However, these methods are unidirectional, testing one locus at a time, resulting in failure to capture the multiple loci controlling complex traits simultaneously. Moreover, multiple test corrections for threshold values are required to control the false positive rate. The Bonferroni correction is often used; however, it is too conservative, resulting in many important loci being ignored because they do not fulfill the significance threshold level [16,17].

Multi-locus models have been proposed as an alternative to overcome the issues with the single-locus model GWAS. These multivariate models consider all loci simultaneously; as a result, multiple test corrections are not needed. So far, several multi-locus GWAS models have been developed and used to study GWAS, such as MLMM (multi-locus mixed-model), FarmCPU (Fixed and random model Circulating Probability Unification), mrMLM (multi-locus random-SNP-effect MLM), FASTmrMLM (fast mrMLM), FASTmrEMMA (fast multi-locus random-SNP-effect efficient mixed model analysis), pLARmEB (polygenic background-control-based least angle regression plus empirical Bayes), pKWmEB (integration of Kruskal–Wallis test with empirical Bayes), ISIS EM-BLASSO (iterative modified-sure independence screening expectation-maximization-Bayesian least absolute shrinkage and selection operator), and GPWAS (Genome-Phenome Wide Association Study) [18]. All the multi-locus models follow the two-step principle during analysis. In the first stage, all the potentially associated SNPs are identified across the whole genome. During the second step, the identified SNPs are included in one model, then their effects are estimated by empirical Bayes, and finally all the non-zero effects are further evaluated using the likelihood ratio test. A less stringent critical *p*-value, such as 0.01, is used to select the SNPs in the first step. Each of these multi-locus model differs in terms of algorithms utilized in the two steps [16,17,19].

Several recent GWAS publications have investigated mineral element concentrations in rice grains using various sets of diverse rice accessions. One study employed 575 rice accessions, including 294 *indica* and 239 *japonica* accessions, largely of Chinese origin, to study 11 minerals in rice grains grown in field trials in China [20]. Another study employed 191 accessions from the USDA mini-core collection, including 100 global *indica* and *aus* and 59 *japonica* accessions, to study 16 minerals in rice grains grown in Beaumont, Texas, of which 9 had significant QTLs detected in the GWAS [18]. Another study focused on 233 accessions of global *indica* germplasm and identified QTLs across 12 mineral elements in brown rice grain harvested in field trials in the Philippines [21]. Although these studies show the success of GWAS to identify key loci controlling mineral concentrations in rice grains, additional studies using diverse sets of germplasm grown across different environments are needed to capture the full range of genetic diversity for these traits.

The objectives of the current study are: (1) to identify loci that are significantly associated with six mineral elements (Cu, Fe, K, Mg, Mn and Zn) by using single-locus and multi-locus GWAS methods using a novel rice diversity panel; and (2) to compare the performance of these methods in terms of detection of trait-associated SNP markers. The findings will accelerate the development of new mineral-rich rice varieties by facilitating marker-assisted breeding (MAB), identifying candidate genes, and providing insight into the molecular mechanisms underlying mineral accumulation in rice grain.

## 2. Materials and Methods

### 2.1. Plant Materials

A total of 174 accessions, including 151 diverse global accessions from the USDA-GRIN germplasm collection and 23 US-released varieties were used in the study. The included accessions flowered in 80–130 days after emergence (DAE) in previous field evaluation to minimize the effect of extremely early and late heading times on rice grain mineral content. These accessions originated from 31 countries, where the highest number of accessions were from Bangladesh (19) followed by Russia (18), Uzbekistan (16), India (14) (Supplementary Table S1).

### 2.2. Sample Preparation for Phenotyping

The field experiment was conducted at the Texas A&M AgriLife Research Center, Beaumont, Texas (30.0802° N, 94.1266° W) in heavy clay soils during from late April to September 2018, which can be considered an average growing season, without any major extremes in weather or conditions. The lines were directly seeded and followed standard practices of keeping a constant flood in the field until all the accessions reached their full maturity along with applying standard fertilizer. A randomized complete block design with two replications with a two-row plot for each replication for each accession was used in the field experiment. Plots were harvested based on individual accession maturity. After reaching the maturity stage, plants in the middle of each plot were bulk harvested and air-dried for 3 months in the drying room. Then, around 120 g of rough seeds were dehulled with electrical dehuller to make brown rice.

### 2.3. Phenotypic Measurements

We used brown rice for mineral content determination for this study. At first, brown rice of all the samples were dried for 72 h at 65 °C, followed by sterilization with 70% ethanol to remove contaminants and/or debris from the surface [22]. Then, seeds were ground into a fine powder by mortar and pestle and kept in airtight plastic zip lock bags or small containers or tubes until the sample digestion was started. A range of 0.5000–0.5002 g of rice sample was weighed accurately and poured directly into MARSXpress digestion vessel (PFA vessel) followed by adding reagents consisting of 6 mL HNO_3_ (12.1 N), and 3 mL of 30% (*v*/*v*) H_2_O_2_ [23]. The digestion vessels were capped and placed in the turntable, followed by heating in the CEM MARS 5 Microwave Accelerated Reaction System (CEM Corporation, NC, USA) using the modified parameters shown in Table 1 [24].

After digestion, the solutions were allowed to cool to room temperature and then were filtered through Whatman No. 1 (11 µm pore size) filter paper into a 25 mL volumetric flask. The volume was brought to the 25 mL mark with ultrapure water. Next, 100× and 1000× diluted samples were prepared from the solution to determine Cu, Fe, Mg and Mn content and K and Zn content, respectively. Inductively Coupled Plasma Mass Spectrometry (ICP-MS) (Agilent Technologies, Santa Clara, CA, USA) was used to quantify the Cu, Fe, K, Mg, Mn and Zn content. Samples were run in total seven batches; each batch composed of three blanks (digestion reagent with no samples), standard reference material (Rice Flour SRM 1568B), and experimental samples were included. Rhodium (Rh 103) was used as internal standard to monitor ICP-MS machine drift while running. During calculation, blanks were averaged and subtracted from experimental samples per batch. Five technical replications were generated for each sample and were averaged. The average elemental concentration of two biological/ field replications of each accession was used during GWAS analysis.

### 2.4. Analysis of Phenotypic Data

Basic statistics, including mean, standard deviation, coefficient of variation (CV), and analysis of variance (ANOVA) were conducted on the whole panel as well as on the two subspecies, *Indica* and *Japonica*, to determine the phenotypic variation. To know the effect of population structure on phenotypic variation, we used ANOVA using the general linear model (GLM), where population structure was set as the fixed variable. In addition, correlation analysis among the minerals was completed. All the analyses were conducted using JMP pro15.

### 2.5. Genotyping

We used 6565 high quality SNPs (SNP calling rate > 0.939; missing data per sample < 6.1%) from the 7K SNP array data [25] for GWAS analysis. To impute the missing genotypes, MACH 1.0 was used, which is a Markov Chain-based haplotyper that infers the missing genotypes by comparing the available genotypes to those in other accessions that have been typed at a higher density [26].

### 2.6. Analysis of Population Structure and Kinship Coefficient

Population structure and kinship analysis were conducted to control the false positive results in the GWAS analysis. STRUCTURE 2.3.4 [27] using Bayesian clustering analysis method was used for determining population structure with the following profile: K, the number of genetic clusters in the panel ranging from 2 to 10 with 10 runs for each K value; burn-in time for each run was 10,000 followed by 50,000 MCMC (Markov Chain Monte Carlo) iterations. The Structure Harvester program (http://taylor0.biology.ucla.edu, accessed on 10 January 2021) was used to determine the best k value using the method of Evanno, Regnaut [28] by submitting the results for each K and determining log(k)2 and ΔK values. Within-population membership probability (Q) threshold was fixed at 0.80, so an individual with higher Q was assigned to a population, whereas an individual having lower Q was considered admixed. For calculating the kinship coefficient matrix (K), four methods implemented in four different software packages were utilized. The TASSEL 5 uses the scaled_IBS method, as a default method, to calculate kinship, whereas the VanRaden method is used in GAPIT. For GEMMA software, a centered relatedness matrix system was used to calculate the kinship in this study. The default method was used during running mrMLM for GWAS analysis.

### 2.7. Linkage Disequilibrium (LD) Analysis

The LD decay distance across the whole genome was measured by squared allele frequency correlations (r^2^) values between the pairs of markers of 6565 SNPs calculated by PopLDdecay 3.41 [29]. Marker pairs were discretized into bins of 1.5 kb, and the average r^2^ value was used as the estimate of r^2^ of a bin. The LD decay was calculated as the chromosomal distance at which the average r^2^ dropped to half of its maximum value [14].

### 2.8. Genome-Wide Association Analysis (GWAS) 

The GWAS was conducted using nine models that can be divided broadly into two groups; single-locus models: CMLM (compressed mixed linear model), ECMLM (Enriched CMLM) and GEMMA (Genome-wide Efficient Mixed Model Association algorithm), and multi-locus models: mrMLM, FASTmrMLM, FASTmrEMMA, pLARmEB, pKWmEB, and ISIS EM-BLASSO. All six multi-locus models are implemented in mrMLM R package [30]. At first, SNPs with *p* < 10^−3^ were considered significant for the single-locus models, and 15% FDR was then applied for the multi-test corrections to declare the final significant SNPs, and these SNPs were used for further analyses. For the multi-locus models, LOD ≥ 3.0 was used as a cut-off value to declare a significant quantitative trait nucleotide (QTN). R^2^ values for significant SNPs were obtained from the respective software used for the GWAS analyses, except for the GEMMA software which does not provide R^2^ values. To calculate R^2^ values for the GEMMA model, we used the following equation:

2 β^2^MAF (1 − MAF)/2β^2^MAF (1 − MAF) + (se(β))^2^ 2N MAF (1 − MAF)

where, β is effect size of genetic variant, MAF is minor allele frequency, se(β) standard error of effect size, and N is sample size [31].

### 2.9. In Silico Gene Expression Analysis

We mined the genes within the LD decay distance on either side of the significant SNPs by using RAP-DB database (https://rapdb.dna.affrc.go.jp/, accessed on 20 January 2021). To check the in silico expression levels of the mined genes, Nipponbare (*japonica*) and IR64 (*indica*) gene expression data were downloaded from the MSU Rice Genome Annotation Project (http://rice.plantbiology.msu.edu/expression.shtml, accessed on 20 January 2021) and the OryzaExpress database (http://riceball.lab.nig.ac.jp/oryzaexpress/, accessed on 20 January 2021), respectively. A heatmap of the gene expression for each trait was created with ComplexHeatmap R package.

## 3. Results

### 3.1. Population Structure and Linkage Disequilibrium (LD)

**Figure 1:**
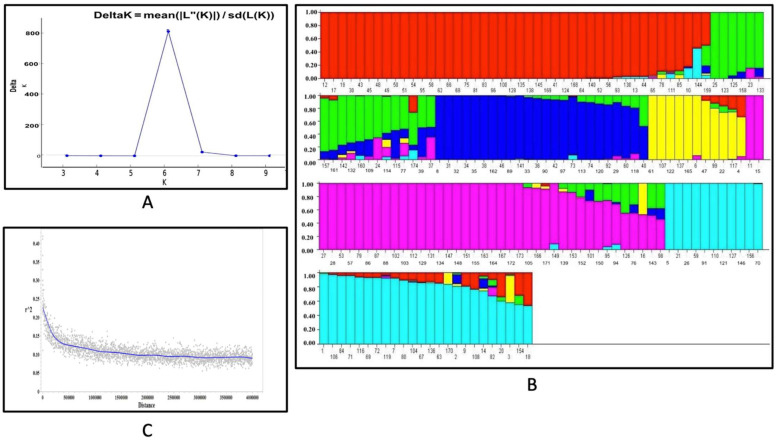
(**A**) Population structure analysis. ΔK is highest at 6, indicating that six groups are present in the rice germplasm used in the study. (**B**) The bar graph displays the result of the STRUCTURE analysis. The bars with different colors show six different sub-populations observed in the diversity panel. (**C**) LD decay distances across the whole genome of rice in this study.

According to the value of Δk from the Structure analysis result, there were six sub-populations in our study sample panel, corresponding to *indica*, *aus*, *aromatic*, *temperate japonica*, and two subgroups of *tropical japonica* genotypes (Figure 1). These six sub-populations were used for the Q-matrix as a covariate during the GWAS analysis to account for the population structure.

**Figure 2:**
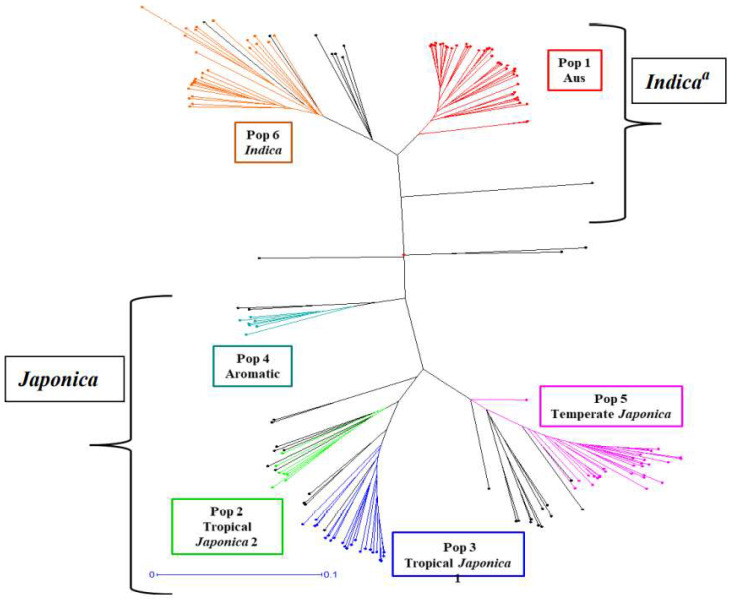
Cluster analysis of the rice germplasm used in this study, using the neighbor-joining method. Colors of the node indicate different subpopulations of the germplasm found by the STRUCTURE analysis. Black color nodes indicate admixed samples. N.B.: a = Subpopulations with close genetic distance are grouped either into “*Indica*” or “*Japonica*” subpopulation, two major subpopulations in rice.

It is well known that rice has two major sub-species, *Indica* and *Japonica*. Studies of global rice germplasm have shown that the *Indica* subspecies consists of the *aus* and *indica* subgroups and the *Japonica* subspecies consists of the *temperate japonica*, *tropical japonica*, and *aromatic* subgroups [32]. To determine the population structure effect on the phenotypic variation, we considered the two primary subpopulations to analyze the phenotypic variation, which was also observed during cluster analysis as two distinct clusters with other sub-groups found within the two main clusters (Figure 2). Three accessions were removed due to admixture. Ultimately, 78 *Indica* accessions and 93 *Japonica* accessions were analyzed in the panel. After genotyping the 171 accessions with 6565 SNP markers using a 7K SNP array, the average linkage disequilibrium (LD) decay across all chromosomes was estimated to be 250 kb, defined as half the maximum of mean r^2^ values (Figure 1).

### 3.2. Phenotypic Variation Analysis

**Figure 3:**
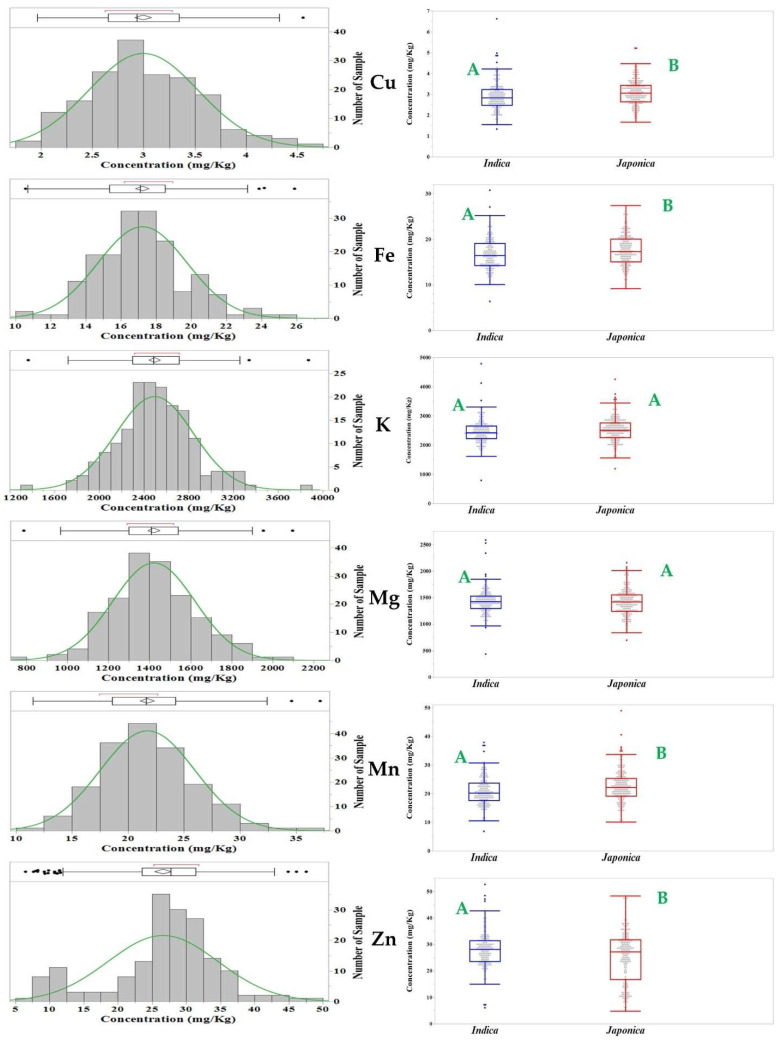
Histogram plots show variation for the concentration of six mineral elements across the rice germplam of this study. Different capital letters (green color) in the box plot indicate that *Indica* and *Japonica* rice accessions are significantly different at α = 0.05 for mean value of the six mineral elements.

The phenotypic evaluation shows a broad variation among accessions. Overall, most traits appeared to be normally distributed, but Zn showed a slightly skewed distribution (Figure 3). Given that the population structure is the main factor affecting GWAS, the population structure explained from 1% (K, Mg) to 10% (Fe) of the phenotypic variation in the whole panel. Mean differences between the *indica* and *japonica* sub-group panels were found to be significant for Cu, Fe, Mn and Zn, but not for K and Mg (Figure 3; Supplementary Table S2). To determine the correlation among the six mineral elements, the Pearson’s correlation coefficients were calculated. All the pairwise correlations between any two minerals were significantly positive and had correlation coefficient (r) values ≥ 0.40, except Mn-Zn, which was significantly positive but r = 0.16 (Supplementary Figure S1).

### 3.3. GWAS Analysis

A SNP was declared as significant using −log_10_ *p* ≥ 3.0 as the first cut-off value for all single-locus models, and LOD ≥ 3.0 for all multi-locus models, respectively. SNPs with MAF < 0.05 were not considered as significant. Multiple SNPs with physical distance of less than 250 kb (the calculated LD) were regarded as the same significant locus (i.e., significant SNP-trait association).

Based on these criteria, a total of 147 significant SNPs for six mineral elements were identified using nine models (Table 2, Supplementary Figures S2 and S3).

**Figure 4:**
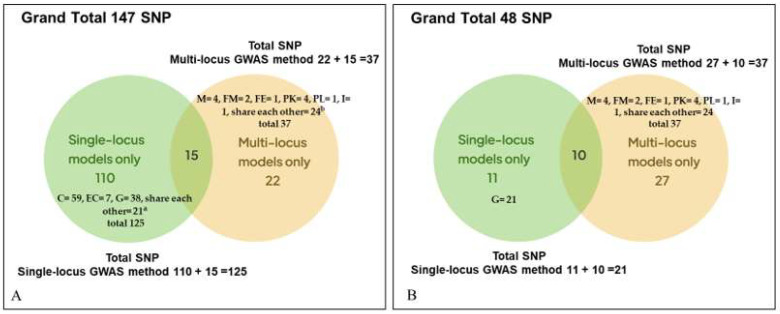
Venn diagrams show the number of SNPs identified by single-locus and multi-locus methods for the six mineral elements (**A**) before and (**B**) after applying multi-test correction. Abbreviations: Single-locus GWAS models-C = CMLM (Compressed Mixed Linear Model); EC = Enhanced Compressed Mixed Linear Model; G = GEMMA (Genome-wide Efficient Mixed Model Association). Multi-locus GWAS models- M = mrMLM (Multi-locus random-SNP-effect MLM); FM = FASTmrMLM (fast mrMLM); FE = FASTmrEMMA (fast multi-locus random-SNP-effect efficient mixed model analysis); PK = pKWmEB (integration of Kruskal–Wallis test with empirical Bayes); PL = pLARmEB (polygenic background-control-based least angle regression plus empirical Bayes); I = ISIS EM-BLASSO (iterative modified-sure independence screening expectation-maximization-Bayesian least absolute shrinkage and selection operator). a = list of shared SNPs among three models; C ∩ EC = 2 SNPs, C ∩ G = 7 SNPs, E ∩ G = 5 SNPs, C ∩ E ∩ G = 7. b = list of shared SNPs among three models; M ∩ FM = 9, M ∩ I = 1, I ∩ PK = 1, FM ∩ I = 1, I ∩ PL = 3, PK ∩ PL = 1, FE ∩ PK = 1, FM ∩ PK ∩ PL = 1, FM ∩ M ∩ PL = 1, FM ∩ M ∩ I = 1, FE ∩ PK ∩ I = 1, I ∩ PK ∩ PL = 1, FM ∩ M ∩ I ∩ PL = 1, M ∩ FM ∩ PK ∩ PL ∩ I = 1.

For Cu concentration, 16 significant SNPs were detected by only single-locus GWAS models and explained 5.91–14.46% of the phenotypic variation. One SNP was found by only multi-locus models, and it explained 5.33–8.69% of the phenotypic variation. Both single and multi-locus models found two additional SNPs that explained 6.97–41.99% of the phenotypic variation. Only single-locus models found 32 SNPs for Fe concentration explaining 6.49–17.89% of the phenotypic variation, whereas only multi-locus models detected two SNPs explaining 3.56–5.33% of the phenotypic variation. Both models identified one SNP for Fe, explaining 6.06 × 10^−10^–26.83% of the phenotypic variation. For K, 13, 7 and 3 SNPs were identified by only single-locus, only multi-locus and both models together, explaining 6.03–23.80%, 3.28–34.37% and 5.86–18.56% of the phenotypic variation, respectively. For Mg, 35 SNPs were found by only single-locus models, explaining 5.83–25.51% of the phenotypic variation. Only two SNPs were detected by only multi-locus, and this explained 8.79–16.40% of the phenotypic variation. Five SNPs were identified by both models that explained 3.62–26.75% of the phenotypic variation. For Mn, single and multi-locus models each detected three SNPs separately, in total six SNPs, that explained 5.94–14.16% and 6.54–10.92% of the phenotypic variation, respectively. For Zn, single-locus, multi-locus and both models identified 11, 7 and 4, in total 22 SNPs, explaining 6.11–49.90%, 5.32–24.44% and 6.15–50.06% of the phenotypic variation. It can be noted that different algorithms per model resulted in different R^2^ per SNP across the different models. However, 147 significant SNPs for all six minerals were reduced to 48 after applying the 15% FDR multi-test correction (Table 2, Figure 4). Of the 48 SNPs, the highest 14 significant SNPs were found for Zn, followed by K (13), Mg (12). Three SNPs each for Cu, Fe, and Mn were found to be significant (Table 2).

**Figure 5:**
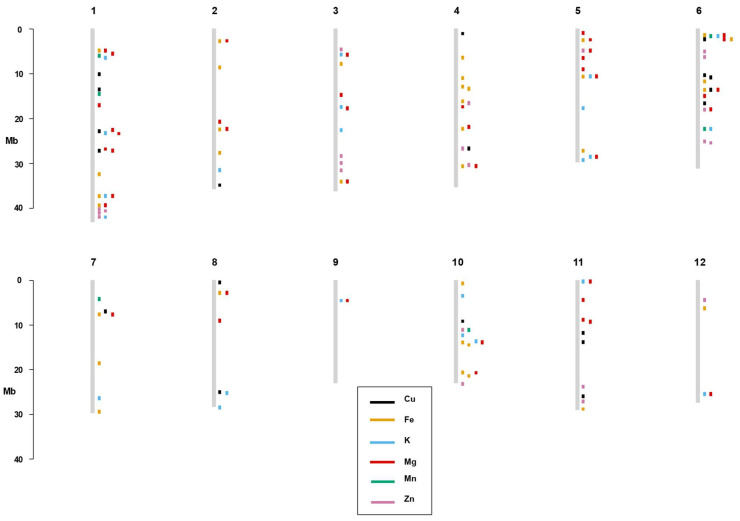
Physical map of the significant SNP markers before applying 15% FDR multi-test correction found in the study for the six mineral elements. SNP positions are depicted by the rectangular box with specific color showing the corresponding mineral elements. Rice chromosomes are displayed by vertical lines.

**Figure 6:**
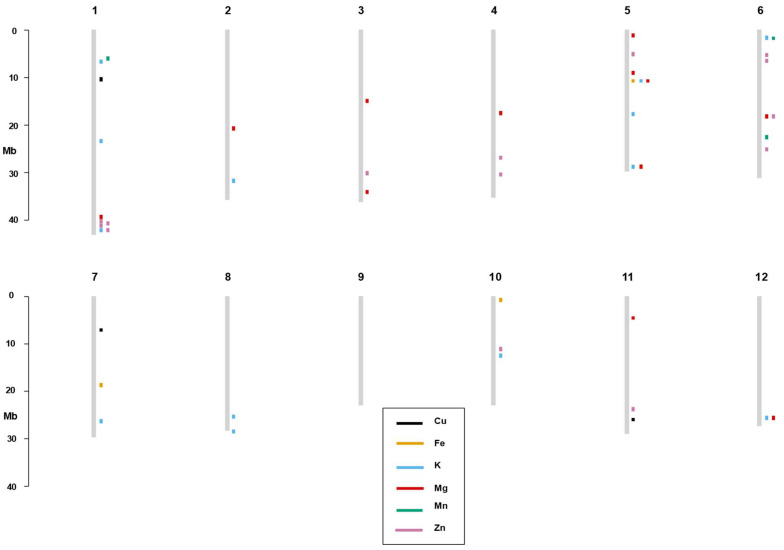
Physical map of the significant SNP markers after applying 15% FDR multi-test correction found in the study for the six mineral elements. SNP positions are depicted by the rectangular box with specific color showing the corresponding mineral elements. Rice chromosomes are displayed by vertical lines.

Among the 147 significant SNPs, 32 SNPs appear to control more than one trait, suggesting a pleiotropic effect. Among the six SNPs that affect Cu, two SNPs (SNP-6.2196821, 6285634) are associated with Fe, three SNPs (907,175, SNP-6.2196821, 6,285,634) with Mg and one SNP (4,572,241) is also found for Zn. Similarly, 15 SNPs have an effect on both Fe and Mg and one SNP on both Fe and K. In addition, seven and one SNPs are associated with controlling both K and Mg and K and Zn, respectively. Two SNPs influence both Mg and Zn elements. Moreover, SNP-6.2196821 and 6,285,634 SNPs are involved in affecting Cu, Fe and Mg, whereas the 1,202,195 SNP has an effect on the Fe, K and Mg elements. Once multi-test correction was applied, 32 SNPs decreased to 18 SNPs. Among the 18 significant SNPs, five SNPs appear to control more than one trait, suggesting a pleiotropic effect. Among the thirteen SNPs that affect K, three SNPs (id5004837, SNP-5.28500625. and 13,022,382) are associated with Mg, and one SNP (SNP-1.41998191.) is found for Zn. Similarly, 6,496,457 SNPs have influence on both Mg and Zn (Table 2, Figure 5 and Figure 6).

In terms of SNP detection ability of the different models used in this study across the six mineral elements, the single-locus GWAS method outperformed the multi-locus GWAS method based on the first level of cut-off value. The single-locus and multi-locus GWAS method identified overall 125 and 37 SNPs, respectively, where 110 and 22 SNPs were identified by single-locus and multi-locus method only, respectively, and both methods shared 15 SNPs (Figure 4A). In addition, in terms of model performance, within single-locus GWAS method, CMLM model detected highest number of SNPs (75 SNPs; CMLM only = 59 SNPs and shared SNPs with other models = 16) and the lowest number of SNPs were identified by ECMLM (21 SNPs; ECMLM only = 7 SNPs and shared SNPs with other models = 14 ) (Figure 4A). Among the six models of multi-locus GWAS method, both mrMLM and FASTmrMLM detected the highest number of SNPs (17 SNPs; mrMLM only = 4 SNPs and shared SNPs with other models = 13; FASTmrMLM only = 2 SNPs and shared SNPs with other models = 15). FASTmrEMMA identified the lowest 3 SNPs (FASTmrEMMA only = 1 SNP and shared with other models = 2). However, the multi-locus GWAS method outperformed the single-locus GWAS method once 15% FDR multi-test correction was applied to the single-locus models. Multi-test correction made the total SNP number of single-locus GWAS method reduced from 125 to 21, in which 10 SNPs were shared, whereas the total SNP number (37 SNPs) was unchanged for Multi-locus GWAS method, where 27 SNPs were belonged to only multi-locus GWAS method (Figure 4B). Multi-test correction also affects the single-locus GWAS model’s performance, where GEMMA all alone identified 21 SNPs (Figure 4B).

### 3.4. In silico Gene Expression Analysis

After mining the genes within 250-kb region of 48 significant SNPs (Figure 4B) using the RAP-DB database (https://rapdb.dna.affrc.go.jp/, accessed on 20 January 2021), we found 43, 36, 273, 197, 85 and 353 genes for Cu, Fe, K, Mg, Mn, and Zn, respectively. To investigate which genes are more likely to be responsible for rice grain mineral traits, we selected only those genes expressed in both reproductive and vegetative stages or only in the reproductive stage by using Nipponbare (*Japonica*) and IR64 (*Indica*) gene expression data in normalized FPKM values. 9, 6, 111, 51, 23, and 98 genes were found to be expressed in Nipponbare, whereas, in IR64, 13, 9, 98, 59, 16, and 116 were expressed for Cu, Fe, K, Mg, Mn, and Zn, respectively, and were used for gene enrichment analysis (Supplementary Figures S4 and S5).

### 3.5. Gene Enrichment Analysis

To understand the function of the expressed genes found in two genetic backgrounds, gene enrichment analysis was carried out in g:Profiler by using Gene Ontology Resources where 5% FDR was used as the significance threshold. As for the background gene list in gene enrichment analysis, all known genes in the *Japonica* genetic background were used for both the *Indica* and *Japonica* gene groups.

For Cu, no similar molecular functions were observed between 9 and 13 expressed genes found in Nipponbare and IR64, but both groups were categorized into three molecular functions. In the case of Fe, 6 expressed genes in *Japonica* were grouped into 15 molecular functions, whereas 9 genes of *Indica* in 30 molecular functions, with six common functional activities. For 111 and 98 expressed genes found in *Japonica* and *Indica* for K, nine molecular functional activities were annotated for each group, with six common functional activities. 59 *Indica* expressed genes for Mg were assigned to 48 molecular functional activities, whereas only one functional activity was found for 51 *Japonica* expressed genes. There were 15 and 16 molecular functional activities observed for 23 *Japonica* and 16 *Indica* expressed genes, respectively, with three common functions, for Mn minerals. For Zn, three and seven GO molecular functional terms were assigned for 98 *Japonica* and 116 *Indica* genetic background expressed genes with one common terms (Supplementary Figure S6).

## 4. Discussion

### 4.1. Population Structure, LD, and Phenotypic Variation

The rice germplasm used in this study has two major sub-populations, *Indica* and *Japonica*, which further split into six sub-populations based on the Structure analysis, which is consistent with the previous studies using worldwide rice germplasm [25,33]. The LD decay distance of this study was 250 kb for the whole panel, which is similar to the previous findings using different sub-populations with LD ranging from 100 kb to over 240 kb for cultivated rice [11]. Mather, Caicedo [34] found LD decay from >500 kb in *Oryza sativa* ssp. *japonica*, to ~75 kb in O. *sativa* subsp. *indica*, and down to merely ~40 kb or lower in O. *rufipogon* for different rice sub-populations. Thus, the LD blocks of this study extend long enough to conduct the association studies using the 7K SNP array, while also providing higher resolution than seen with biparental mapping populations.

Sufficient phenotypic variation for all the mineral traits used in this study was observed, suggesting that GWAS can be applied to this rice diversity panel. Positive correlations with moderate levels were observed among the six mineral elements except between Mn and Zn, indicating that these minerals might share common gene regulation pathways. This could also be due to the pleiotropic effects of causal genes controlling these minerals in rice, which is supported by our GWAS findings: 32 pleiotropic SNPs were found at our first significance threshold level and five were detected at the second significance level, potentially explaining the correlation observed among the minerals. While no pleiotropic SNPs were found between Mn and Zn, a positive correlation with a low level was found in correlation studies, indicating SNPs with minor effects still might exist that our GWAS could not capture.

### 4.2. Performance of Single and Multi-Locus GWAS Models

In terms of SNP detection ability, based on our GWAS studies, single-locus models altogether found more significant loci (125 SNPs) than multi-locus models (37 SNPs) at the first significant cut-off value, but its number reduced to 21 once a 15% FDR multiple test correction was applied to the single-locus models. Multiple test correction is not required for multi-locus methods, which is an obvious advantage, so the number of SNPs detected by these methods will be same. Therefore, the performance of the multi-locus models was better compared to the single-locus models in our study. Similar results were also reported in the previous studies using both real and simulation datasets where the multi-locus approach was more powerful than the single-locus approach [16,17,35]. However, Liu et al. (2020) detected more SNPs with univariate models (GLM and MLM) than with multivariate models (mrMLM and FarmCPU), which is similar to our result of the first significant cut-off value. Having a smaller number of potential causal SNPs with the multi-test correction in our study may be due to not having enough sample size and failing to capture the small effects associated with complex traits [19]. Regarding model performances, GEMMA is believed to be the best single-locus model in this study, where it identified second highest SNPs (57 SNPs) after CMLM and was the only representative single-locus model once multi-test correction applied. As for six multi-locus GWAS models, except FASTmrEMMA, both mrMLM and FASTmrMLM were the highest SNP-identifying models (17 SNPs) and the remaining three models performed similarly, detecting around 10 SNPs.

As the main goal of this study is to find as many significant SNPs as possible without losing any potential causal SNPs, we applied two of three approaches necessary to determine stable QTLs: one was applying multiple approaches by using single and multi-locus GWAS models and another was verification by previously reported QTLs, which will be described in the next section [35]. By applying the first approach, 48 significant SNPs are being reported as reliable in this study because they were detected across several multi-locus models or by single-locus plus multi-locus models. Thus, this study supports the previous recommendation by applying both single-locus and multi-locus models to get stable, reliable QTLs [18]. In addition, we recommend to include the GEMMA model, combined with multi-locus models, in any future GWAS studies of complex traits.

### 4.3. Comparison and Reliability of Our GWAS Studies

To evaluate the reliability of our QTLs, we compared our detected SNPs for the six minerals with the genes/QTLs and markers related to mineral content identified from previous linkage mapping and association mapping studies. To compare with the QTL/marker locations of the previous studies, the surrounding 250 kb of our associated SNPs were regarded as potentially the same locus for any particular trait when this region was found between the borders of previously identified QTL/marker locations. In the case of SNP marker comparisons, the markers of past studies that were located within the 250 kb region of our significant associated SNPs were considered as the same locus for the particular trait. Thus, with these parameters, 43 (~29%) of the 147 of the significant SNPs identified by the first significance threshold of this study coincided with previously reported genes/QTLs and/or markers for the six minerals and the remaining 104 (70%) SNPs could be considered as novel. Out of the verified 43 SNPs, 17 were found for Fe, followed by eight for Mg, seven for K, five for both Cu and Zn and the remaining one was found for Mn. However, after applying a multiple test correction to the single-locus models, 43 significant SNPs that verified by co-location with previously reported QTLs now reduced to 14 verified SNPs (~29%), supporting the fact that the multiple test correction can eliminate true QTLs identified in the single-locus GWAS study. Out of the established 14 SNPs, 5 SNPs were detected for K, followed by 3 for Zn and 2 SNPs each for Cu ang Mg. Both Fe and Mn had one SNP (Supplementary Table S3).

The molecular mechanisms of uptake, transport and accumulation of the mineral elements used in this study are well established [36]. So far, several genes and gene families have been found to be involved in the acquisition and transport of copper and zinc in rice seeds. These include, but are not limited to, the ZIP (Zinc-regulated transporter (ZRT)) gene families, Iron-regulated transporter (IRT-like protein) gene family, YSL (yellow stripe-like) protein, MTPs (metal tolerance proteins), COPT (COPper Transporter) family, and NRAMPs gene family. Some of them are also involved in the pathway of uptake, transport, and accumulation of iron, magnesium and cadmium [37]. ZIP3 of the six ZIP genes (ZIP1, 3, 4, 5, 7a and 8) was identified within a 250 kb region at id4010220 SNP in chromosome 4 associated with Zn in our GWAS study. Two SSRs and five SNPs were also reported in the same chromosomal position by [36,38,39]. Similarly, the *NAS* gene family is involved in the accumulation of Fe, Zn and Cu in rice endosperm [40]. The current study found *OsNAS3* at SNP-7.29385457. in chromosome 7 associated with Fe at our first threshold value for the significance test, where two SSR markers were also reported before [3,38], but it was removed once multiple test correction was applied. The *COPT* transporter gene family for Cu was not identified by our GWAS analysis. Interestingly, a known gene, *OsIRO2*, an iron-related bHLH transcription factor 2 that regulates Fe uptake from soil, transport during germination, and translocation to the grain, was co-located at 1,305,247 SNP in chromosome 1 associated with Zn in our study, where Bollinedi et al. (2020) also found a SNP at almost the same position, supporting the fact that a single gene may control the molecular mechanism of multiple elements simultaneously (Supplementary Table S3).

The fact that 14 QTLs, two of which co-localized with known genes, were rediscovered by this study, supports the accuracy of our GWAS study. More importantly, these 14 QTLs regulating six mineral elements, simultaneously detected in various populations with different genetic backgrounds, eventually can be further validated and used to conduct marker-assisted selection in future biofortification programs.

### 4.4. Functional Annotation of Candidate Genes

The experimental validation of candidate loci/QTLs disclosed by mapping studies is essential for marker-assisted selection (MAS) and other plant development programs [17]. Although further experiments will be required to prove the causal genes controlling mineral concentrations in the rice grain, we nevertheless tried to better understand the molecular mechanisms of those six mineral traits by examining candidate genes in those regions. In this study, 298 and 311 genes were identified in the *japonica* and *indica* genetic background, respectively, located within a 250-kb region of the significant SNPs associated with six mineral elements and expressed beyond the vegetative stage, suggesting that the two major rice sub-species might have different genetic pathways controlling those mineral traits.

To better understand the biological function of those expressed genes, we further conducted gene enrichment analysis in g:Profiler using gene ontology (GO). For Cu, the analysis revealed that the genes were significantly enriched at 5% FDR in three and four molecular function GO terms for *Japonica* and *Indica*, respectively, without sharing any common function. The genes in *indica* genetic background were categorized as mainly antiporter and transporter activity of K and Na, whereas *japonica* genes were involved in structural molecule activity and binding (drug and cyclosporin). The genes for Fe in *japonica* and *indica* were classified into 15, and 30 molecular function GO terms with 14 common functions between them. The common functions mostly consist of activity (cation, proton, and kinase) and binding (such as anion, carbohydrate derivative, nucleoside phosphate, ribonucleotide, etc.). For K, nine molecular function terms were assigned for each group of genes with eight common functions, revealing that both genomes could follow similar molecular pathways for K. However, for Mg, the two genomes might have different molecular mechanisms because 48 molecular functional terms were identified for *indica* genes, whereas only one term was assigned for *japonica* genes. Among the 48 functions, some functions are well known for Mg roles, for example, DNA-binding transcription activator activity, RNA polymerase II-specific, NADPH binding, and catalytic activity, indicating the possibility that some candidate genes in the *indica* genetic background might not be present in *japonica*. For Mn, 15 and 16 molecular function terms were enriched for the *japonica* and *indica* genes, with three common functions between them that were mostly involved in mannosyltransferase activity. For Zn, the *japonica* and *indica* genes were enriched into three and seven molecular functional terms, with one common between them (Figure S6). Overall, the functional annotation elucidated the similarity and differences in molecular mechanisms responsible for mineral content used by two major rice populations. These results can be used to design future studies for functional gene characterization and to select appropriate genetic backgrounds for gene cloning.

A potential limitation of this study is using one year/location data that cannot provide assurance of the stability of the significant SNPs identified in our study. However, we tried to validate our findings by comparing with previous studies, showing 29% of our significant SNPs co-located with the previously known gene/markers. Future research can be used to further validate our findings by (1) conducting additional GWAS studies in multiple locations and years and (2) using gene-editing tools to characterize the candidate genes identified in this study.

## 5. Conclusions

This study reported the GWAS of six mineral elements using 174 global rice accessions and 7k SNP array genotype data. A total of 48 SNPs affecting mineral elements were identified by single-locus and multi-locus methods. GEMMA along with five multi-locus models (mrMLM, FASTmrMLM, pKWmEB, pLARmEB, and ISIS EM-BLASSO) were the best models to identify significant SNP in the single-locus and multi-locus GWAS method, respectively, used in this study. While 14 SNPs matched with previously reported genes/QTLs and markers, 34 SNPs were novel. After mining genes within a 250 kb region of these SNPs, a total of 987 genes were found. Among these genes, 298 and 311 genes in *japonica* and *indica*, respectively, were used for enrichment analysis to know their potential functions. Thus, they could be identified as candidate genes for controlling accumulation in rice grain. These shortlisted genes could be used for future studies to further investigate the gene expression levels, followed by functional gene characterization, to better understand the complex molecular mechanisms controlling rice grain concentration of these six mineral elements.

## Figures and Tables

**Table 1:** Parameters used during microwave digestion.

Power (W)	% Max	Time (min) to Raise Temperature (℃)	Temperature	Running Time (min)
1600	50	20.5	160	4.5

**Table 2:** List of significant loci detected in the study at −log_10_P ≥ 3.0 and 15% FDR cut-off value for Single-locus GWAS, and LOD ≥ 3.0 cut-off value for Multi-locus GWAS.

Trait	SNP	Alleles	Chr.	Pos.(bp)	Single-Locus GWAS			Multi-Locus GWAS		
Cu					−log10P	R^2^	Model	LOD	R^2^	Model
	304,900 ^a^	G/A	1	10,100,605	4.07–4.55	8.37–17.62	C, EC, G	3.84–6.82	9.38–20.57	**M, FM, PK, PL, I** ^b^
	SNP-1.13478728.	G/A	1	13,479,755	3.55	10.04	G			
	760,644	A/C	1	22,723,681	3.57	7.81	G			
	907,175	G/A	1	27,047,209	3.67	7.28	G			
	id2015767	A/G	2	34,868,096	3.50	7.20	G			
	id4000574	C/A	4	972,749	3.22	9.12	C			
	4,572,241	G/A	4	26,688,183	3.02, 3.23	7.20, 13.60	C, EC			
	SNP-6.2196821.	A/T	6	2,197,821	3.01	8.16	G			
	6,147,112	G/A	6	10,199,497	3.01	5.91	G			
	SNP-6_10761128.	A/G	6	10,762,128	3.47	6.93	G			
	6,285,634	G/A	6	13,487,635	3.21	9.08	C			
	6,427,131	A/G	6	16,508,748	3.40	9.73	G			
	id7001155	G/A	7	6,987,625	3.37–3.91	6.97–15.91	C, EC, G	3.30	41.99	**M**
	7,993,541	C/A	8	416,250	3.51	7.46	G			
	id8006885	G/A	8	24,753,844	3.21	9.10	C			
	SNP-10.9068762.	G/A	10	9,139,902	3.17	8.96	C			
	11,233,430	G/A	11	11,715,177	3.06	13.70	EC			
	SNP-11.13313880.	G/A	11	13,777,560	3.35	14.46	EC			
	SNP-11.25392640.	A/C	11	25,858,722				3.25, 4.16	5.33, 8.69	**I, PK**
Fe	153,297	G/A	1	4,823,701	3.77	10.55	C			
	SNP-1.32376151.	G/A	1	32,377,196	3.48	9.71	C			
	1,202,195	A/C	1	37,230,818	3.16, 3.21	6.60, 8.77	C, G			
	1,257,104	A/G	1	39,282,883	3.24	12.81	G			
	SNP-2.2575985.	G/A	2	2,575,988	3.17	8.79	C			
	SNP-2.8455563.	G/C	2	8,455,565	3.38	6.64	G			
	2,087,054	A/G	2	22,324,900	3.15	8.75	C			
	2,267,750	A/C	2	27,548,893	3.25	9.05	C			
	id3004190	A/G	3	7,849,199	3.02	8.38	C			
	3,501,392	G/A	3	33,987,612	3.45	6.77	G			
	SNP-4.6317262.	G/A	4	6,321,823	3.28	6.49	G			
	SNP-4.10930754.	A/G	4	10,940,054	3.08	8.54	C			
	4,128,471	C/A	4	12,879,859	3.10	8.59	C			
	SNP-4.13348501.	C/A	4	13,357,791	3.28	9.12	C			
	4,241,771	G/A	4	16,199,670	3.54	9.87	C			
	SNP-4.22128339.	G/A	4	22,313,458	3.66	10.22	C			
	4,678,550	G/A	4	30,601,123	4.28	12.08	C			
	4,882,140	A/C	5	2,378,143	3.23	8.97	C			
	5,196,119	A/G	5	10,528,231	3.36–3.52	6.97–18.43	C, EC, G	3.47–3.99	6.06 × 10^−10^–26.83	**FM, PK, PL**
	5,735,083	A/G	5	27,094,485	3.17–3.47	6.89–17.89	C, EC, G			
	SNP-6.1343132.	A/G	6	1,344,132	3.38	9.41	C			
	SNP-6.2196821.	A/T	6	2,197,821	3.14	8.70	C			
	id6007260	A/G	6	11,618,178	3.13	8.68	C			
	6,285,634	G/A	6	13,487,635	3.86	10.82	C			
	7,179,219	A/G	7	7,619,494	3.37	9.37	C			
	7,643,802	G/A	7	18,573,822				3.67, 4.34	4.94, 5.33	**FM, I**
	SNP-7.29385457.	A/G	7	29,386,450	3.02	8.38	C			
	8,067,129	G/A	8	2,887,584	3.25, 3.60	6.71, 10.07	C, G			
	9,921,984	A/G	10	650,031				3.53	3.56	**FM**
	SNP-10.13843768.	T/A	10	13,915,001	3.31	9.21	C			
	10,555,828	G/A	10	14,438,582	3.09	8.56	C			
	SNP-10.20587837.	G/A	10	20,659,359	3.13	8.70	C			
	10,778,744	A/G	10	21,397,933	3.16	17.88	EC			
	SNP-11.28200021.	C/G	11	28,723,243	3.29	9.14	C			
	SNP-12.6356528.	C/A	12	6,357,639	3.73	10.44	C			
K	SNP-1.6382810.	G/A	1	6,383,811	3.06	8.62	C	3.22	14.41	**M**
	SNP-1.23170758.	G/A	1	23,171,803				3.70	34.37	**PK**
	1,202,195	A/C	1	37,230,818	3.37, 3.75	6.59, 23.80	EC, G			
	SNP-1.41998191.	T/A	1	41,999,235				3.90–5.05	5.27–8.37	**FM, I, M**
	2,375,486	G/A	2	31,547,627				3.01	10.55	**M**
	SNP-3.5666296.	G/A	3	5,667,297	3.10	8.74	C			
	2,964,807	G/A	3	17,453,008	3.03	6.02	G			
	3,173,191	A/G	3	22,657,915	3.31	6.56	G			
	id5004837	G/A	5	10,539,124	3.00, 4.06	6.57, 8.44	C, **G**			
	5,452,087	A/G	5	17,598,723				3.33, 3.67	7.05, 11.58	**FM, M**
	SNP-5.28500625.	C/G	5	28,563,271	4.32	18.56	**G**	3.11, 4.40	5.69, 12.04	**FM, M**
	5,787,299	A/G	5	29,150,826	3.12, 3.30	6.03, 22.76	EC, G			
	SNP-6_1500959.	A/G	6	1,501,961				3.44	7.69	**PK**
	6,674,186	C/A	6	22,249,886	3.06	7.61	G			
	7,892,688	G/A	7	26,282,546				3.78, 4.51	4.92, 8.06	**FM, M**
	8,966,923	C/A	8	25,213,697				3.24, 3.30	3.28, 3.85	**I, M**
	id8007977	A/G	8	28,377,609	3.31	6.59	**G**	3.19	5.86	**FM**
	c9p4565514	C/A	9	4,565,515	3.15	6.09	G			
	10,063,204	A/G	10	3,400,212	3.10	6.13	G			
	10,480,545	G/A	10	12,310,115	3.15, 4.27	6.97, 8.81	C, **G**			
	id10003608	G/A	10	13,711,367	3.09	8.70	C			
	SNP-11.235195.	G/A	11	236,194	3.04	8.57	C			
	13,022,382	A/C	12	25,490,919	3.00, 4.06	6.57, 8.45	C, **G**			
Mg	153,297	G/A	1	4,823,701	3.19	8.82	C			
	170,435	G/A	1	5,408,523	3.34	9.28	C			
	572,891	G/A	1	17,008,280	3.11	8.62	C			
	id1012746	A/G	1	22,494,508	3.16	6.14	G			
	SNP-1.23342685.	G/A	1	23,343,730	2.98	8.22	C			
	899,561	A/G	1	26,762,494	3.10	6.71	C			
	907,175	G/A	1	27,047,209	3.16	6.13	G			
	1,202,195	A/C	1	37,230,818	3.02–3.29	5.83–25.51	C, EC, G			
	1,257,104	A/G	1	39,282,883	3.98	9.82	**G**	3.16, 3.42	3.62, 4.18	**I, PL**
	SNP-2.2575985.	G/A	2	2,575,988	3.05	8.42	C			
	2,031,305	G/A	2	20,616,529	4.13	10.43	**G**			
	2,087,054	A/G	2	22,324,900	3.03	8.38	C			
	SNP-3.5666296.	G/A	3	5,667,297	3.63	10.12	C			
	2,853,978	G/A	3	14,652,096				3.17	16.40	**M**
	2,972,375	G/A	3	17,674,269	3.08	8.50	C			
	3,501,392	G/A	3	33,987,612	3.71	7.40	**G**			
	4,288,833	A/G	4	17,316,219	3.39	6.96	G	3.79, 3.80	5.49, 7.13	**I, PL**
	4,448,877	G/A	4	21,864,875	4.60	13.00	C			
	4,678,550	G/A	4	30,601,123	3.36	9.34	C			
	4,833,352	A/G	5	922,530	3.52–3.85	7.51–26.75	C, EC, **G**	4.84–7.43	7.65–13.28	**FM, I, M, PL**
	4,882,140	A/C	5	2,378,143	3.05	8.44	C			
	id5002528	C/A	5	4,819,475	3.35	9.30	C			
	5,011,602	G/A	5	6,374,926	3.21	8.88	C			
	5,121,882	A/G	5	8,842,405				3.00–4.02	4.90–12.68	**FM, M, PL**
	id5004837	G/A	5	10,539,124	3.32, 3.77	7.30, 7.48	C, **G**			
	SNP-5.28500625.	C/G	5	28,563,271	3.93	8.55	**G**			
	SNP-6.1343132.	A/G	6	1,344,132	3.09	8.55	C			
	SNP-6.2196821.	A/T	6	2,197,821	3.31	9.18	C			
	6,285,634	G/A	6	13,487,635	3.69	10.30	C			
	6,351,040	G/A	6	14,937,819	3.45	9.58	C			
	6,496,457	C/A	6	17,969,922	3.50	7.80	**G**	3.50, 4.80	4.84, 8.31	**FM, M**
	7,179,219	A/G	7	7,619,494	3.53	9.82	C			
	8,067,129	G/A	8	2,887,584	3.62	10.07	C			
	8,322,255	G/A	8	9,019,202	3.28	9.09	C			
	c9p4565514	C/A	9	4,565,515	3.27	6.40	G			
	SNP-10.13843768.	T/A	10	13,915,001	4.10	11.51	C			
	SNP-10.20587837.	G/A	10	20,659,359	3.21	8.89	C			
	SNP-11.235195.	G/A	11	236,194	3.04	8.41	C			
	10,943,015	A/G	11	4,419,880	3.06, 3.99	8.11, 24.99	EC, **G**	3.62, 4.04	7.65, 8.59	**I, PL**
	11,112,426	G/A	11	8,788,201	3.12	10.39	G			
	11,130,199	G/A	11	9,217,367	3.12	10.39	G			
	13,022,382	A/C	12	25,490,919	3.32–3.77	7.3–7.48	C, **G**			
Mn	SNP-1.5867020.	A/G	1	5,868,021				3.42–3.68	10.92–20.24	**FE, I, PK**
	SNP-1.14460354.	G/A	1	14,461,381	3.25	14.16	EC			
	5,868,825	A/C	6	1,521,855				4.01	6.54	**PK**
	SNP-6.22337184.	G/A	6	22,338,182				3.31	4.87–7.88	**FM, M**
	7,066,952	G/A	7	4,232,489	3.10	5.94	G			
	id10002943	C/A	10	11,195,773	3.05	13.66	EC			
Zn	1,280,193	A/G	1	40,154,802	3.76, 4.34	8.87, 49.90	EC, **G**			
	SNP-1.40596823.	T/A	1	40,597,867	3.48, 3.86	7.92, 49.46	EC, **G**	6.08	23.93	**FE**
	1,305,247	G/A	1	41,042,727	3.66–4.74	9.73–50.06	C, EC, **G**	3.03–6.73	8.61–11.01	**I, PK, PL**
	SNP-1.41998191.	T/A	1	41,999,235				4.22	6.01	**PL**
	SNP-3.4621271.	G/A	3	4,622,270	3.26	6.89	G			
	SNP-3.28426789.	G/A	3	28,433,737	3.04	6.45	G			
	3,405,830	G/A	3	29,987,079				4.78	16.90, 24.44	**FM, M**
	rd3001044	A/G	3	31,627,459	3.00	6.11	G			
	id4004654	G/A	4	16,559,384	3.17	8.77	C			
	4,572,241	G/A	4	26,688,183	3.92	7.80	**G**			
	id4010220	A/G	4	30,330,971				3.44	7.40	**I**
	id5002528	C/A	5	4,819,475	4.05	50.34	EC	3.18	6.65, 13.02	**FM, M**
	rd6001756	G/A	6	5,007,776	4.17	9.26	**G**			
	SNP-6.6241072.	A/G	6	6,242,072				3.31	6.00, 11.38	**FM, M**
	6,496,457	C/A	6	17,969,922				3.08–3.47	5.32, 7.43	**PK, PL**
	SNP-6.25063527.	G/A	6	25,064,525	3.01	6.15	**G**	7.36	10.14, 14.37	**FM, M**
	6,783,797	G/A	6	25,387,111	3.00, 3.88	6.44, 50.08	C, EC			
	10,430,775	A/G	10	11,051,662				5.96	12.63	**PK**
	id10007301	A/G	10	23,033,344	3.66	10.18	C			
	11,769,276	G/A	11	23,660,957				3.85, 8.13	5.35, 14.00	**FE, PK**
	11,915,122	G/A	11	27,015,384	3.65	49.72	EC			
	12,134,336	G/A	12	4,433,511	3.04	8.39	C			

a = The underlined SNP names were declared significant at 15% FDR and LOD ≥ 3.0 significant threshold level for Single-locus GWAS method and Multi-locus GWAS method, respectively. b = Models name with bold identified those underlined SNPs at 15% FDR and LOD ≥ 3.0 significance threshold level for Single-locus GWAS method and Multi-locus GWAS method, respectively. Abbreviations: Single-locus GWAS models-C = CMLM (Compressed Mixed Linear Model); EC = Enhanced Compressed Mixed Linear Model; G = GEMMA (Genome-wide Efficient Mixed Model Association). Multi-locus GWAS models- M = mrMLM (Multi-locus random-SNP-effect MLM); FM = FASTmrMLM (fast mrMLM); FE = FASTmrEMMA (fast multi-locus random-SNP-effect efficient mixed model analysis); PK = pKWmEB (integration of Kruskal–Wallis test with empirical Bayes); PL = pLARmEB (polygenic background-control-based least angle regression plus empirical Bayes); I = ISIS EM-BLASSO (iterative modified-sure independence screening expectation-maximization-Bayesian least absolute shrinkage and selection operator).

## Data Availability

All data are available in the Supplementary Materials.

## Supplementary Materials

The following are available online at: https://www.mdpi.com/article/10.3390/genes13122330/s1, Table S1: Description of the 174 rice genotypes analyzed in the study, Table S2: Phenotypic variation in the whole panel and *Indica* and *Japonica* sub-groups, Table S3: Comparison of the GWAS result with the previous studies, Figure S1: Correlation matrix for the six mineral elements, Figure S2: Manhattan plots of GWAS for six minerals, Figure S3: Physical map for significant SNP at −log10P ≥ 3.0 significant threshold value for six mineral elements, Figure S4: Heatmap of In silico gene expression analysis results for the six mineral elements of the study in Nipponbare, Figure S5: Heatmap of In silico gene expression analysis results for the six mineral elements of the study in IR64, Figure S6. Gene enrichment analysis result, depicting potential molecular functional GO terms for (1). Cu, (2). Fe, (3). K, (4). Mg, (5). Mn and (6). Zn.
