# Supplementary material for: Identifying the Genetic Basis of Mineral Elements in Rice Grain Using Genome-Wide Association Mapping
Source: Genes (Basel). 2022 Dec 10;13(12):2330. doi: 10.3390/genes13122330 (PMC9777918; doi:10.3390/genes13122330)
Supplement: Supplementary file 1 [file genes-13-02330-s001.zip › genes-1964874-supplementary.pdf]

Supplementary Materials

Table S1. Description of the 174 rice genotypes analyzed in the study.

Sample	Plant_ID	Name	Origin	Subpopulations of the study	Sample order in STRUCTURE[a]	Sample classification in two major subpopulations[b]
RG-2	CIor 12153	Quinimpol	Philippines	Admixture	115	*Japonica*
RG-4	CIor 12234	Long Gnar Jim	US	Pop 3/Tropical *Japonica*-1	92	*Japonica*
RG-6	CIor 12244	Creole Bred	US	Admixture	39	*Japonica*
RG-9	CIor 2490	Karang Serang	Indonesia	Admixture	77	*Japonica*
RG-11	CIor 7404	Kin Shan Zim	China	Pop 6/*Indica*	84	*Indica*
RG-14	CIor 9403	Century Patna Original	US	Pop 3/Tropical *Japonica*-1	29	*Japonica*
RG-15	PI 127076	Spin Mere	Afghanistan	Pop 1/Aus	135	*Indica*
RG-18	PI 160530	Pan Ju	China	Pop 6/*Indica*	110	*Indica*
RG-19	PI 161567	Criollo Chivacoa 2	Venezuela	Admixture	40	*Japonica*
RG-21	PI 180060	Dhala Shaitta	Bangladesh	Pop 1/Aus	44	*Indica*
RG-23	PI 199553	Secano do Brazil	El Salvador	Pop 2/Tropical *Japonica*-2	125	*Japonica*
RG-24	PI 208447	Early No. 1	Nepal	Pop 6/*Indica*	59	*Indica*
RG-28	PI 223612	Sel. No. 388	Uruguay	Admixture	126	*Japonica*
RG-29	PI 224605	Sigadis	Indonesia	Pop 5/Temperate *Japonica*	131	*Japonica*
RG-30	PI 226204	SHIMIZU MOCHI	Japan	Pop 5/Temperate *Japonica*	129	*Japonica*
RG-31	PI 229262	N 32	India	Pop 1/Aus	100	*Indica*
RG-33	PI 238190	Charmarumi	India	Pop 1/Aus	30	*Indica*
RG-34	PI 240638	Dular	India	Pop 1/Aus	54	*Indica*
RG-37	PI 277414	Red Khosha Cerma	Afghanistan	Admixture	117	*Japonica*

RG-39	PI 283681	Hashikalmi Aus	Bangladesh	Pop 1/Aus	66	*Indica*
RG-40	PI 283682	Kataktara Aus	Bangladesh	Pop 1/Aus	81	*Indica*
RG-42	PI 291608	WC 4443	Bolivia	Admixture	174	Admixture
RG-43	PI 294423	GHRAIBA	Iraq	Pop 1/Aus	65	*Indica*
RG-44	PI 297569	Dharial	Bangladesh	Pop 1/Aus	45	*Indica*
RG-48	PI 369813	Samanis	Suriname	Pop 2/Tropical *Japonica*-2	123	*Japonica*
RG-49	PI 373053	Bala	India	Admixture	18	*Indica*
RG-51	PI 373232	Khao Phoi	Laos	Admixture	82	*Indica*
RG-52	PI 373347	Karayal	Sri Lanka	Pop 1/Aus	78	*Indica*
RG-53	PI 373403	ARC 6578	India	Pop 1/Aus	10	*Indica*
RG-55	PI 373537	ARC 10638	India	Pop 6/*Indica*	9	*Indica*
RG-56	PI 373777	C 8447	Indonesia	Pop 2/Tropical *Japonica*-2	25	*Japonica*
RG-57	PI 373779	Tia Heret	Indonesia	Admixture	142	*Japonica*
RG-60	PI 373816	Padi Pohon Batu	Malaysia	Admixture	109	*Japonica*
RG-62	PI 376252	Pelu	India	Pop 1/Aus	111	*Indica*
RG-65	PI 385344	Ratna	India	Pop 6/*Indica*	116	*Indica*
RG-67	PI 385529	Jhona	Pakistan	Pop 1/Aus	75	*Indica*
RG-68	PI 385578	Sufaida	Pakistan	Pop 1/Aus	138	*Indica*
RG-70	PI 385621	Mahlar	Pakistan	Pop 1/Aus	96	*Indica*
RG-73	PI 385849	Ziri	Pakistan	Pop 1/Aus	168	*Indica*
RG-74	PI 385888	Sathra	Pakistan	Pop 1/Aus	124	*Indica*
RG-75	PI 389037	Ai Chueh Ta Pai Ku	Taiwan	Pop 6/*Indica*	1	*Indica*
RG-77	PI 389267	Heo Trang	Vietnam	Pop 6/*Indica*	67	*Indica*
RG-78	PI 389876	Sipirasikkam	Indonesia	Pop 2/Tropical *Japonica*-2	133	*Japonica*
RG-79	PI 389879	Sigoendaba	Indonesia	Admixture	132	*Japonica*

RG-80	PI 389945	Angkrang	Cambodia	Pop 6/*Indica*	7	*Indica*
RG-81	PI 389960	Srav Prapay	Cambodia	Pop 6/*Indica*	136	*Indica*
RG-83	PI 391827	Lantjang	Indonesia	Pop 6/*Indica*	91	*Indica*
RG-84	PI 391936	Ali Combo	Madagascar	Pop 6/*Indica*	5	*Indica*
RG-85	PI 391943	Sabharaj	Bangladesh	Pop 6/*Indica*	121	*Indica*
RG-86	PI 392170	Torh	Pakistan	Admixture	144	*Indica*
RG-87	PI 392217	Sugdasi	Pakistan	Pop 1/Aus	140	*Indica*
RG-90	PI 392677	ASWINA 330	Bangladesh	Pop 1/Aus	13	*Indica*
RG-92	PI 393114	DNJ 151	Bangladesh	Pop 1/Aus	52	*Indica*
RG-94	PI 400042	AS 46	India	Pop 1/Aus	12	*Indica*
RG-95	PI 400586	Putih Montor	Indonesia	Admixture	114	*Japonica*
RG-96	PI 400587	Gendjah Banten	Indonesia	Pop 6/*Indica*	63	*Indica*
RG-98	PI 400662	Janeri	Nepal	Pop 6/*Indica*	72	*Indica*
RG-101	PI 400773	Vary Vato 275	Madagascar	Admixture	154	*Indica*
RG-105	PI 400782	Bengaly Morino 120	Madagascar	Admixture	20	*Indica*
RG-107	PI 401750	Kuning Tinggi	Indonesia	Pop 3/Tropical *Japonica*-1	89	*Japonica*
RG-109	PI 402634	Koi Murali	India	Pop 1/Aus	85	*Indica*
RG-111	PI 402691	Trandeup Kandir	Cambodia	Pop 1/Aus	145	*Indica*
RG-112	PI 402720	Angana	India	Pop 1/Aus	169	*Indica*
RG-113	PI 402747	Banajira	Bangladesh	Pop 1/Aus	19	*Indica*
RG-114	PI 402804	Brondol	Indonesia	Admixture	24	*Japonica*
RG-117	PI 403091	DJ 53	Bangladesh	Pop 1/Aus	49	*Indica*
RG-118	PI 403109	DJ 90	Bangladesh	Pop 1/Aus	50	*Indica*
RG-119	PI 403114	DJ 102	Bangladesh	Pop 1/Aus	48	*Indica*
RG-120	PI 403160	DM 55	Bangladesh	Pop 1/Aus	51	*Indica*

RG-121	PI 403287	DV 85	Bangladesh	Pop 1/Aus	56	*Indica*
RG-123	PI 403310	DV 132	Bangladesh	Pop 1/Aus	55	*Indica*
RG-130	PI 412790	Daudzai Field Mix	Pakistan	Pop 1/Aus	41	*Indica*
RG-137	PI 413802	Bengawan	Indonesia	Pop 6/*Indica*	21	*Indica*
RG-138	PI 413989	Sug	India	Pop 5/Temperate *Japonica*	139	*Japonica*
RG-148	PI 431292	Akabona	Pakistan	Pop 6/*Indica*	2	*Indica*
RG-150	PI 433833	Aus 8	Bangladesh	Admixture	14	*Indica*
RG-154	PI 439078	Ngoba	India	Pop 6/*Indica*	104	*Indica*
RG-159	PI 494105	M202	US	Admixture	94	*Japonica*
RG-160	PI 497682	IR64	Philippines	Pop 6/*Indica*	71	*Indica*
RG-178	PI 575134	Bak Tushi	Bangladesh	Pop 1/Aus	17	*Indica*
RG-179	PI 575201	Gambir	Bangladesh	Pop 1/Aus	62	*Indica*
RG-181	PI 575212	Ghorbhai	Bangladesh	Pop 1/Aus	64	*Indica*
RG-182	PI 575217	Shoni	Bangladesh	Pop 1/Aus	130	*Indica*
RG-186	PI 584569	FIROOZ	Iran	Pop 4/Aromatic	61	*Japonica*
RG-189	PI 584625	Ak Tokhum	Azerbaijan	Admixture	4	Admixture
RG-192	PI 585042	EMBRAPA 1200	Brazil	Pop 3/Tropical *Japonica*-1	60	*Japonica*
RG-193	PI 593892	Jefferson	US	Pop 3/Tropical *Japonica*-1	74	*Japonica*
RG-226	PI 67150	Mushkan	India	Pop 4/Aromatic	99	*Japonica*
RG-237	PI 231642	Caucasica	Russia	Pop 5/Temperate *Japonica*	27	*Japonica*
RG-257	PI 277417	Shevkati Kundry	Azerbaijan	Pop 1/Aus	128	*Indica*
RG-259	PI 282171	ARPA SHALI	Uzbekistan	Pop 5/Temperate *Japonica*	11	*Japonica*
RG-263	PI 282208	Uz Rosz 17	Uzbekistan	Pop 5/Temperate *Japonica*	151	*Japonica*
RG-264	PI 282210	UZ ROSZ 269	Uzbekistan	Pop 5/Temperate *Japonica*	147	*Japonica*
RG-266	PI 282212	Uz Rosz 2832	Uzbekistan	Admixture	152	*Japonica*

RG-269	PI 291427	Uz Rosz M9	Uzbekistan	Pop 5/Temperate *Japonica*	153	*Japonica*
RG-276	PI 346926	Nahodka	NA	Admixture	101	*Japonica*
RG-277	PI 346927	VILKID ZIRE	NA	Pop 5/Temperate *Japonica*	155	*Japonica*
RG-279	PI 346932	KUBAN 3	Russia	Pop 5/Temperate *Japonica*	88	*Japonica*
RG-280	PI 348904	Uz Ros 275	Uzbekistan	Pop 5/Temperate *Japonica*	149	*Japonica*
RG-283	PI 348909	SADRI MASALINSKIJ	Azerbaijan	Pop 4/Aromatic	122	*Japonica*
RG-284	PI 348910	Ambarby White	Azerbaijan	Pop 4/Aromatic	6	*Japonica*
RG-288	PI 373900	Besudi Long-Grain	Afghanistan	Admixture	22	*Japonica*
RG-289	PI 373901	Besudi Short-Grain	Afghanistan	Pop 2/Tropical *Japonica*-2	23	*Japonica*
RG-305	PI 431000	Dera Wadi 1/43	Afghanistan	Pop 1/Aus	43	*Indica*
RG-310	PI 431024	Cat 1747	Russia	Pop 6/*Indica*	26	*Indica*
RG-311	PI 431031	P 817	Russia	Admixture	108	*Indica*
RG-316	PI 431195	Vulgaris Ko Ch Azpasaly	Uzbekistan	Pop 6/*Indica*	156	*Indica*
RG-319	PI 431201	UZ ROS 59	Uzbekistan	Pop 6/*Indica*	146	*Indica*
RG-332	PI 431235	P 1041	Russia	Pop 6/*Indica*	106	*Indica*
RG-334	PI 431242	P 1048	Russia	Pop 4/Aromatic	107	*Japonica*
RG-336	PI 431267	HZ ROS 637	Uzbekistan	Pop 6/*Indica*	69	*Indica*
RG-338	PI 439621	Azerbaidjanica	Azerbaijan	Pop 5/Temperate *Japonica*	15	*Japonica*
RG-340	PI 439624	Kasakstanica	Kazakhstan	Pop 6/*Indica*	80	*Indica*
RG-343	PI 439629	Nigrescens	Russia	Pop 5/Temperate *Japonica*	105	*Japonica*
RG-346	PI 439633	Ak Tohum	Azerbaijan	Admixture	3	*Indica*
RG-348	PI 439637	Dicolorata	Azerbaijan	Pop 4/Aromatic	47	*Japonica*
RG-354	PI 439650	Bak Saly Mestnyj	Azerbaijan	Admixture	16	*Japonica*
RG-355	PI 439661	DONSKOJ 2	Russia	Pop 5/Temperate *Japonica*	53	*Japonica*
RG-357	PI 439664	Dv Ros 0219	Russia	Pop 5/Temperate *Japonica*	57	*Japonica*

RG-358	PI 439665	Dv Ros 2568	Russia	Pop 1/Aus	58	*Indica*
RG-363	PI 439677	Kasaki Shala Mestnyj	Uzbekistan	Pop 5/Temperate *Japonica*	79	*Japonica*
RG-364	PI 439679	Kesa	Azerbaijan	Pop 6/*Indica*	170	*Indica*
RG-365	PI 439683	KUBANETS 508	Russia	Pop 5/Temperate *Japonica*	171	*Japonica*
RG-371	PI 439724	Severnyj	Russia	Pop 5/Temperate *Japonica*	172	*Japonica*
RG-372	PI 439730	UZBEKSKIJ 2	Uzbekistan	Pop 5/Temperate *Japonica*	148	*Japonica*
RG-373	PI 439733	Uz Ros 421	Uzbekistan	Admixture	150	*Japonica*
RG-375	PI 446913	Pioner 320	Uzbekistan	Pop 5/Temperate *Japonica*	112	*Japonica*
RG-376	PI 458444	Krasnodarski	Russia	Pop 5/Temperate *Japonica*	86	*Japonica*
RG-379	PI 584584	LUK TAKHAR	Afghanistan	Pop 1/Aus	93	*Indica*
RG-380	PI 584615	WIR 623	Uzbekistan	Pop 5/Temperate *Japonica*	166	*Japonica*
RG-383	PI 584618	WIR 1528	Azerbaijan	Pop 5/Temperate *Japonica*	163	*Japonica*
RG-384	PI 584620	Hi Muke	Kazakhstan	Pop 1/Aus	68	*Indica*
RG-385	PI 584622	WIR 2623	Russia	Pop 5/Temperate *Japonica*	164	*Japonica*
RG-389	PI 584629	Celiaj	Azerbaijan	Pop 5/Temperate *Japonica*	28	*Japonica*
RG-390	PI 584633	UZ ROS 2759	Uzbekistan	Pop 5/Temperate *Japonica*	173	*Japonica*
RG-393	PI 584637	KROS 358	Kazakhstan	Pop 5/Temperate *Japonica*	87	*Japonica*
RG-394	PI 584640	NF-1	Russia	Pop 5/Temperate *Japonica*	102	*Japonica*
RG-395	PI 584642	NF-9	Russia	Pop 5/Temperate *Japonica*	103	*Japonica*
RG-396	PI 584644	SPALCIK	Russia	Pop 5/Temperate *Japonica*	134	*Japonica*
RG-400	PI 584649	INTENSIVNYJ	Uzbekistan	Pop 6/*Indica*	70	*Indica*
RG-403	PI 584652	ZEMCYZNYJ	Russia	Pop 5/Temperate *Japonica*	167	*Japonica*
RG-404	PI 596813	WIR 3419	Azerbaijan	Pop 4/Aromatic	165	*Japonica*
RG-412	PI 61718	Shala	Turkistan	Pop 6/*Indica*	127	*Indica*
RG-413	PI 65884	Styk	Azerbaijan	Pop 4/Aromatic	137	*Japonica*

RG-550	PI 636839	WAB450-11-1-3-P40-HB	Cote D'Ivoire	Pop 2/Tropical *Japonica*-2	157	*Japonica*
RG-551	PI 636840	WAB450-24-2-3-P33-HB	Cote D'Ivoire	Pop 2/Tropical *Japonica*-2	158	*Japonica*
RG-552	PI 636841	WAB450-24-3-P38-1-HB	Cote D'Ivoire	Admixture	159	Admixture
RG-557	PI 636846	WAB450-I-B-P-38-HB	Cote D'Ivoire	Admixture	160	*Japonica*
RG-558	PI 636847	WAB450-I-B-P-62-HB	NA	Admixture	161	*Japonica*
RG-575	PI 636465	Presidio	US	Pop 3/Tropical *Japonica*-1	113	*Japonica*
RG-578	PI 385751	Kharsu	Pakistan	Pop 1/Aus	83	*Indica*
U-117	NA	JAZZMAN 2	US	Pop 3/Tropical *Japonica*-1	73	*Japonica*
U-118	NA	CL 172	US	Pop 3/Tropical *Japonica*-1	36	*Japonica*
U-119	NA	M206	US	Admixture	95	*Japonica*
U-120	NA	CL 163	US	Pop 3/Tropical *Japonica*-1	35	*Japonica*
U-158	NA	DELLA 2	US	Pop 3/Tropical *Japonica*-1	42	*Japonica*
U-159	NA	ANTONIO	US	Pop 3/Tropical *Japonica*-1	8	*Japonica*
U-160	NA	THAD	US	Pop 3/Tropical *Japonica*-1	141	*Japonica*
U-17	NA	CL 111	US	Pop 3/Tropical *Japonica*-1	32	*Japonica*
U-18	NA	CL 153	US	Pop 3/Tropical *Japonica*-1	34	*Japonica*
U-199	NA	RONDO	US	Pop 6/*Indica*	119	*Indica*
U-20	NA	MERMENTAU	US	Pop 3/Tropical *Japonica*-1	97	*Japonica*
U-200	NA	CL 151	US	Pop 3/Tropical *Japonica*-1	33	*Japonica*
U-37	NA	JUPITER	US	Admixture	76	*Japonica*
U-38	NA	WELLS	US	Pop 3/Tropical *Japonica*-1	162	*Japonica*
U-39	NA	LAKAST	US	Pop 3/Tropical *Japonica*-1	90	*Japonica*
U-40	NA	DIAMOND	US	Pop 3/Tropical *Japonica*-1	46	*Japonica*
U-56	NA	MM-14	US	Admixture	98	*Japonica*
U-57	NA	REX	US	Admixture	118	*Japonica*

U-58	NA	CHENIERE	US	Pop 3/Tropical *Japonica*-1	31	*Japonica*
U-59	NA	COCODRIE	US	Pop 3/Tropical *Japonica*-1	38	*Japonica*
U-60	NA	CL272	US	Admixture	37	*Japonica*
U-79	NA	ROY J	US	Pop 3/Tropical *Japonica*-1	120	*Japonica*
U-80	NA	TITAN	US	Admixture	143	*Japonica*

a= STRUCTURE software assigned serial number, called “Unit”, to each sample used in this study. These numbers were displayed in Figure 1(B); b= Based on cluster analysis (Figure 2), subpopulations with close genetic distance were grouped either into “*Indica”* or “*Japonica”* subpopulation, two major subpopulations in rice. These two major groups had been used for phenotypic analysis, *In-Silico* Gene Expression analysis, and Gene enrichment analysis.

Table S2. Phenotypic variation in the whole panel and *Indica* and *Japonica* sub-groups.

Traits	Whole Panel							Indica		Japonica	
	Mean	SD	Min	Max	CV	$R^{2\ a}$	P-value	Mean	SD	Mean	SD
Cu	2.99	0.66	1.33	6.63	22.06	0.02	0.04	2.91*	0.70	3.06	0.62
Fe	17.21	3.45	6.37	30.77	20.04	0.10	0.00	16.71*	3.43	17.64	3.42
K	2492.84	422.67	793.67	4788.57	16.96	0.01	0.34	2471.84	425.71	2510.32	420.47
Mg	1419.99	253.32	435.10	2585.49	17.84	0.01	0.11	1434.59	250.75	1407.83	255.47
Mn	21.78	5.38	6.85	48.99	24.71	0.02	0.02	20.88*	5.04	22.54	5.55
Zn	26.53	8.77	4.75	52.64	33.05	0.04	0.00	27.96*	6.81	25.34	9.98

* indicates that mean mineral concentration of *Indica and Japonica* subpopulation was significantly different from each other at α= 0.05 level.

a= R^2 indicates how much variation of mineral element concentration is explained by population structure in one-way ANOVA model.

Table S3. Comparison of the GWAS result with the previous studies.

Trait	SNP	Position (bp)	Chr	Markers linked/ associated	Position (bp)	Types of Markers	Known genes	References
Cu	SNP-1.13478728.	13479755	1	ud1000606	13429671	SNP		Norton et al. (2014)
	6147112	10199497	6	id6006288	10090472	SNP		Norton et al. (2014)
	id7001155**	6987625	7	RM214	5Mb - 20Mb	SSR		Zhang et al. (2014)
	id8006885	24881549	8	RM3155, id8007452	23Mb – 28Mb, 27210520	SSR, SNP		Zhang et al. (2014), Norton et al. (2014)
	SNP-11.25392640. **	25858722	11	id11010366, id11010372, id11010373	25850373, 25851231, 25851251	SNP		Norton et al. (2014)
Fe	153297	4823701	1	RM283	4886944 - 4886983	SSR		Nawaz et al. (2015)
	SNP-1.32376151.	32377196	1	RM5	23Mb - 35Mb	SSR		Zhang et al. (2014)
	SNP-2.8455563.	8455565	2	RG437, RM452, RM145/Os02ssr0079000	7Mb – 1Mb0, 7706972 - 7707033	RFLP, SSR, SSR		Zhang et al. (2014), Nawaz et al. (2015)
	2267750	27548893	2	RM6933	24Mb - 31Mb	SSR		Zhang et al. (2014)
	3501392	33987612	3	RM514, AX-95935621, AX-95950999, AX-95935460, AX-95924055, AX-95923317, AX-95923159	29Mb – 36Mb, 32326592, 32335075, 32374286, 32380341, 32380432, 32380964	SSR, SNP		Zhang et al. (2014), Bollinedi et al. (2020),
	4241771	16199670	4	RM3317, RM471	5Mb – 20Mb, 18996850 - 18996873	SSR, SSR		Zhang et al. (2014), Nawaz et al. (2015)
	SNP-4.6317262.	6321823	4	RM3317	5Mb - 20Mb	SSR		Zhang et al. (2014)
	SNP-4.10930754.	10940054	4	RM3317	5Mb - 20Mb	SSR		Zhang et al. (2014)
	4128471	12879859	4	RM3317	5Mb - 20Mb	SSR		Zhang et al. (2014)
	SNP-4.13348501.	13357791	4	RM3317	5Mb - 20Mb	SSR		Zhang et al. (2014)
	4882140	2378143	5	RM13	0 - 5500000	SSR		Zhang et al. (2014)

	SNP-6.1343132.	1344132	6	RM190, RM190	0 – 4Mb, 1765669 - 1765704	SSR, SSR		Zhang et al. (2014), Nawaz et al.(2015)
	SNP-6.2196821.	2197821	6	RM190	0 - 4Mb	SSR		Zhang et al. (2014)
	SNP-7.29385457.	29386450	7	RM248, RM1335	26Mb – 30Mb, 28299722 - 28299763	SSR, SSR	OsNAS3	Zhang et al. (2014), Nawaz et al.(2015)
	9921984**	650031	10	RM222	0 - 12Mb	SSR		Zhang et al. (2014)
	SNP-10.20587837.	20659359	10	RM1108	18Mb - 21Mb	SSR		Zhang et al. (2014)
	10778744	21397933	10	RM1108	18Mb - 21Mb	SSR		Zhang et al. (2014)
K	SNP-1.6382810. **	6383811	1	RG532a, RM1	3Mb - 7Mb	RFLP, SSR		Zhang et al. (2014)
	SNP-1.23170758. **	23171803	1	CDO455, RM5501, RM5	23Mb – 35Mb, 23972466 - 23972495	RFLP, SSR		Zhang et al. (2014), Garcia-Oliveira et al (2009)
	2375486**	31547627	2	RM3732-RM492	7290000 - 44080000	SSR		Du et al. (2013)
	2964807	17453008	3	RM282-RM6266/Os03ssr0099800-Os03ssr0183400	12408722 - 23823856	SSR		Du et al. (2013)
	3173191	22657915	3	RM282-RM6266/Os03ssr0099800-Os03ssr0183400	12408722 - 23823856	SSR		Du et al. (2013)
	SNP-5.28500625. **	28563271	5	RM188	21Mb - 27Mb	SSR		Zhang et al. (2014)
	7892688**	26282546	7	RM248, RM505/RM21926	26Mb – 30Mb, 24527811 - 24527834	SSR		Zhang et al. (2014), Nawaz et al.(2015)
Mg	4882140	2378143	5	RM13	0 - 5500000	SSR		Zhang et al. (2014)
	id5002528	4819475	5	RM13	0 - 5500000	SSR		Zhang et al. (2014)
	6285634	13487635	6	RM527-RM3	9862309 - 28130383	SSR		Du et al. (2013)
	6351040	14937819	6	RM527-RM3	9862309 - 28130383	SSR		Du et al. (2013)
	6496457**	17969922	6	RM527-RM3	9862309 - 28130383	SSR		Du et al. (2013)
	SNP-10.20587837.	20659359	10	RM5494	18Mb - 21Mb	SSR		Zhang et al. (2014)

	SNP-11.235195.	236194	11	RM5494	18Mb - 21Mb	SSR		Zhang et al. (2014)
	10943015**	4419880	11	RZ781, RM332	0 - 5Mb	RFLP, SSR		Zhang et al. (2014)
Mn	SNP-6.22337184. **	22338182	6	RM527-RM3	9862309 - 28130383	SSR		Du et al. (2013)
Zn	1305247**	41042727	1	AX-95918225	41121295	SNP	OsIRO2	Bollinedi et al. (2020)
	SNP-3.28426789.	28433737	3	id3013232	28309539	SNP		Norton et al. (2014)
	id4010220**	30330971	4	RM317, id4010984, wd4003179, id4011016, id4011022, AX-95951158, RM317/Os04ssr0174300	28Mb – 33Mb, 31853012, 31953475, 31953961, 31957137, 32811874, 29246223 - 29246242	SSR, SNP	ZIP3	Zhang et al. (2014), Norton et al. (2014), Bollinedi et al. (2020), Huang et al.(2015)
	10430775**	11051662	10	RM222, id10003681, AX-95932094	0 – 12Mb, 13867606, 12685215	SSR, SNP		Zhang et al. (2014), Norton et al. (2014), Bollinedi et al. (2020)

** indicates the significant SNPs identified at 15% FDR.

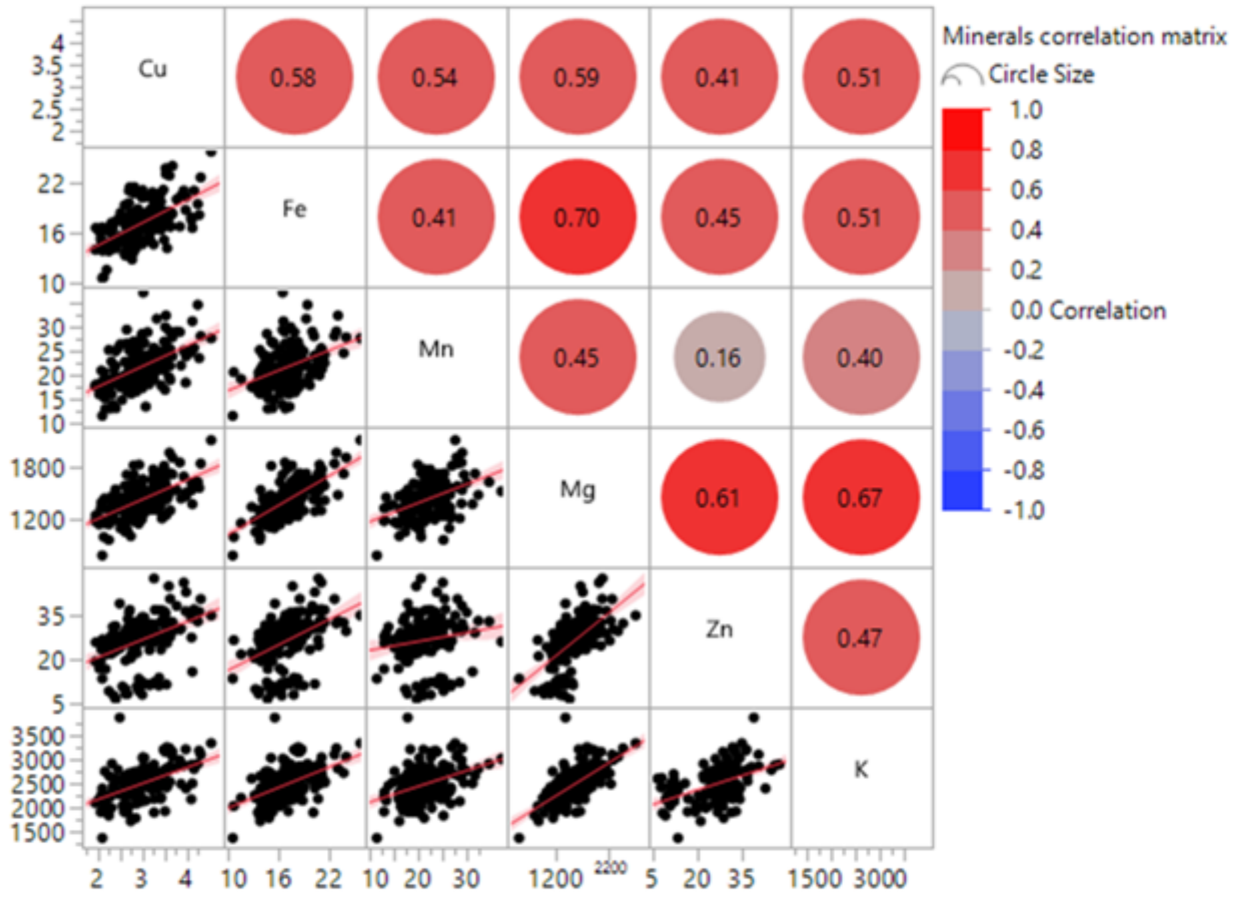

Figure S1. Correlation matrix for the six mineral elements. The value inside the circles shows correlation value between two minerals. Size of the circle indicate the magnitude of significant level at α= 0.05.

(A)

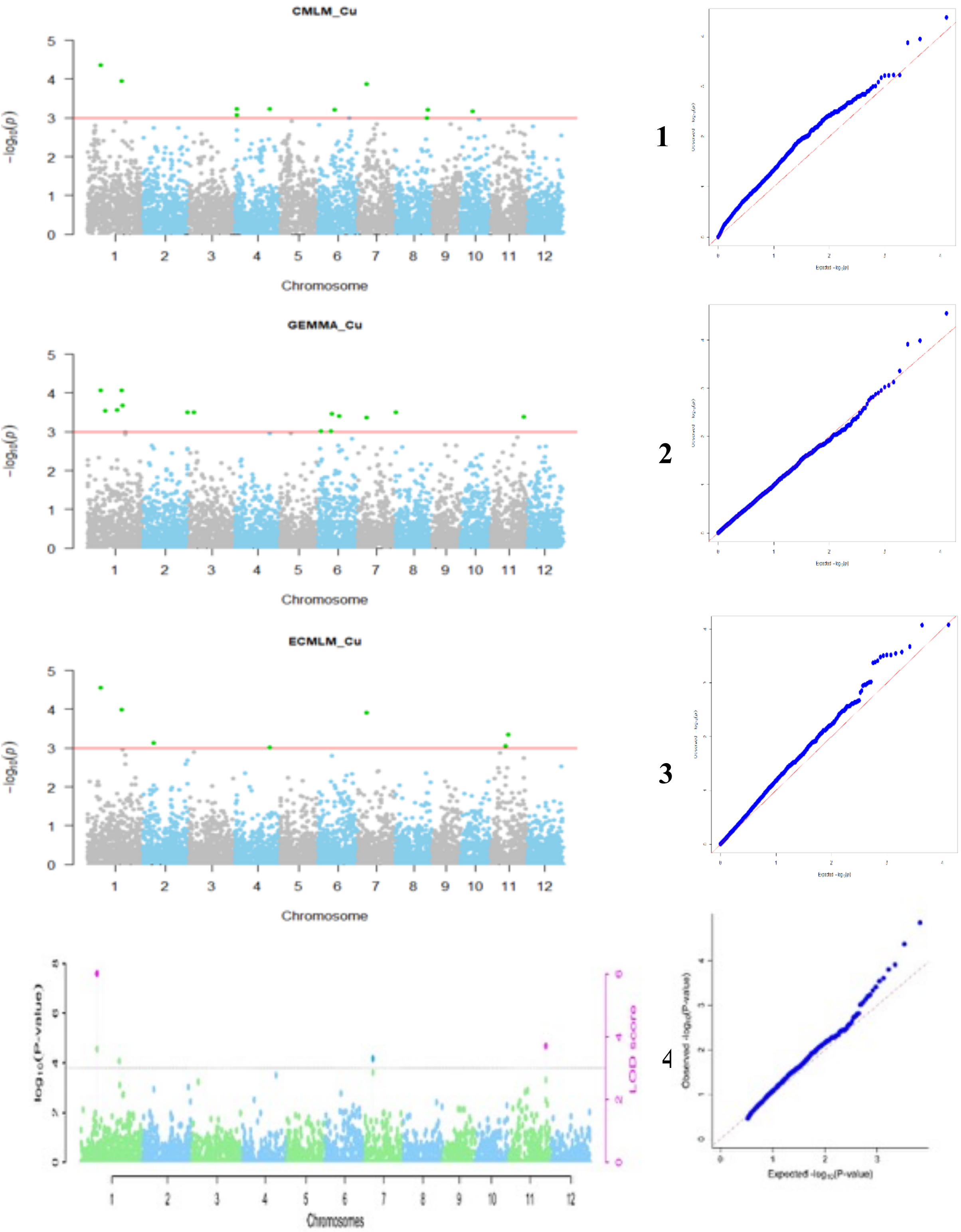

CMLM_Cu
GEMMA_Cu
ECMLM_Cu
Chromosome
Chromosomes
LOD score
Observed -log10(P-value)
Expected -log10(P-value)
1
2
3
4

(B)

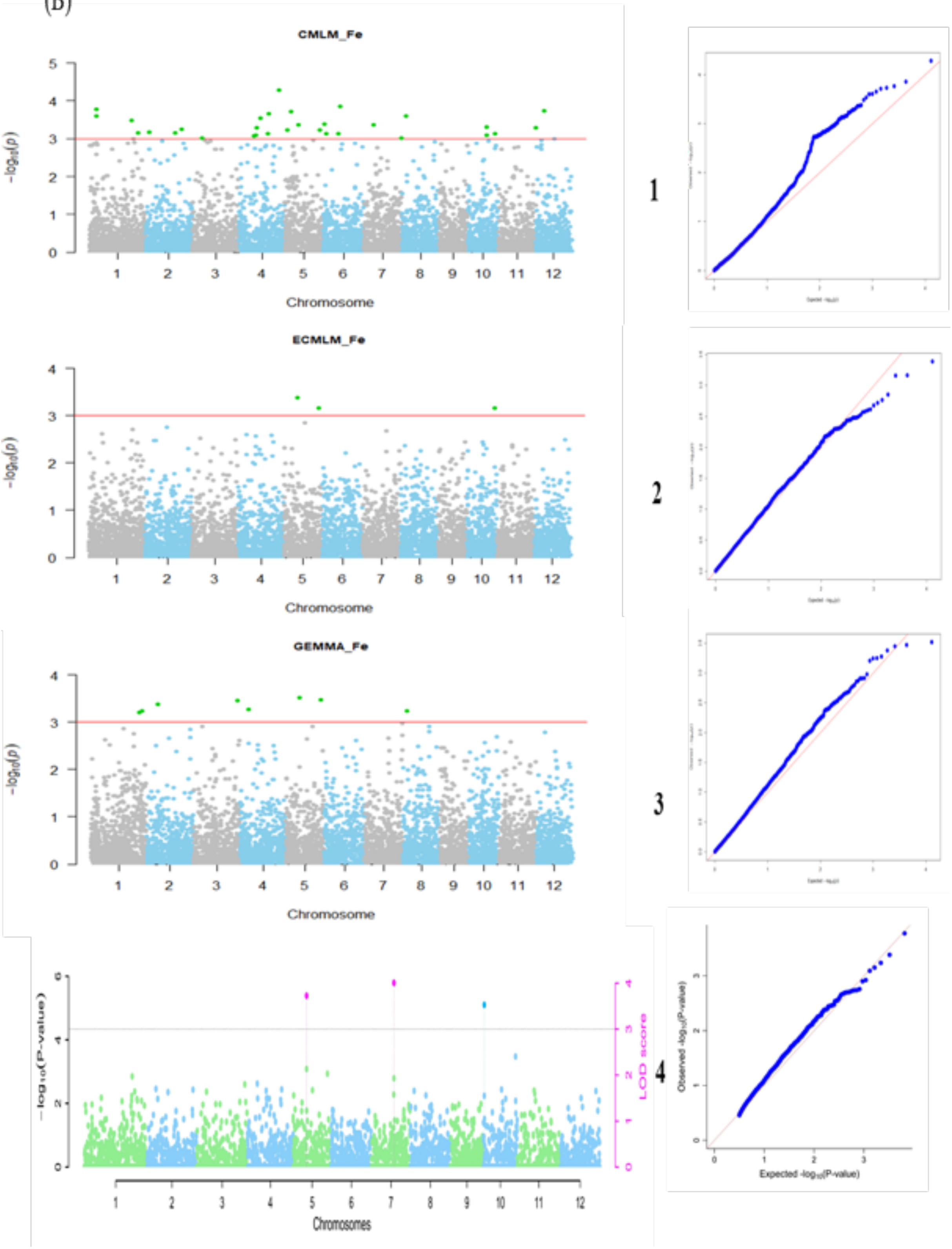

CMLM_Fe
ECMLM_Fe
GEMMA_Fe
$-\log_{10}(p)$
Chromosome
$-\log_{10}$(P-value)
LOD score
Chromosomes
Observed $-\log_{10}$(P-value)
Expected $-\log_{10}$(P-value)
1
2
3
4

(C)

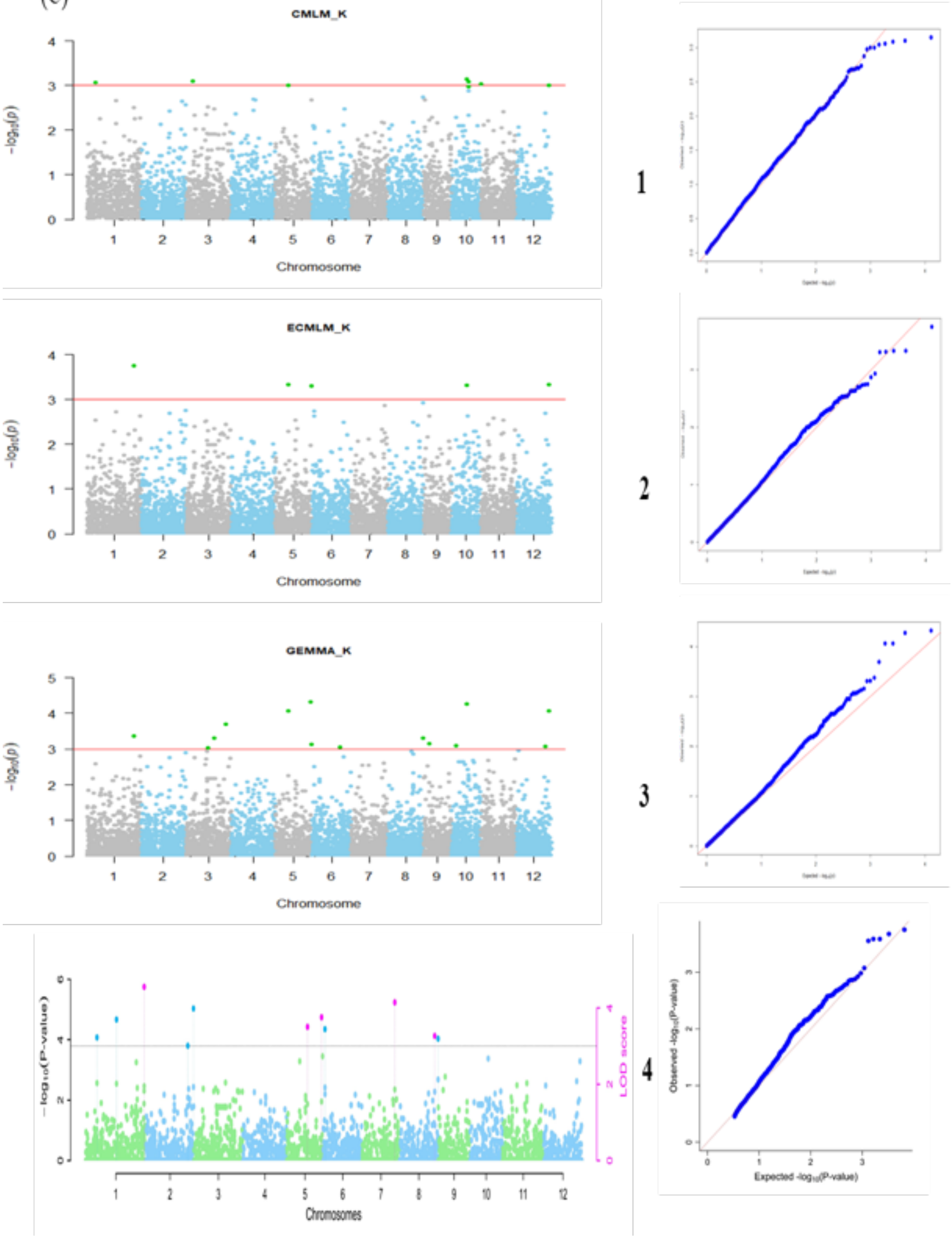

CMLM_K
ECMLM_K
GEMMA_K
$-\log_{10}(p)$
Chromosome
$-\log_{10}$(P-value)
LOD score
Chromosomes
1
2
3
4
Observed $-\log_{10}$(P-value)
Expected $-\log_{10}$(P-value)

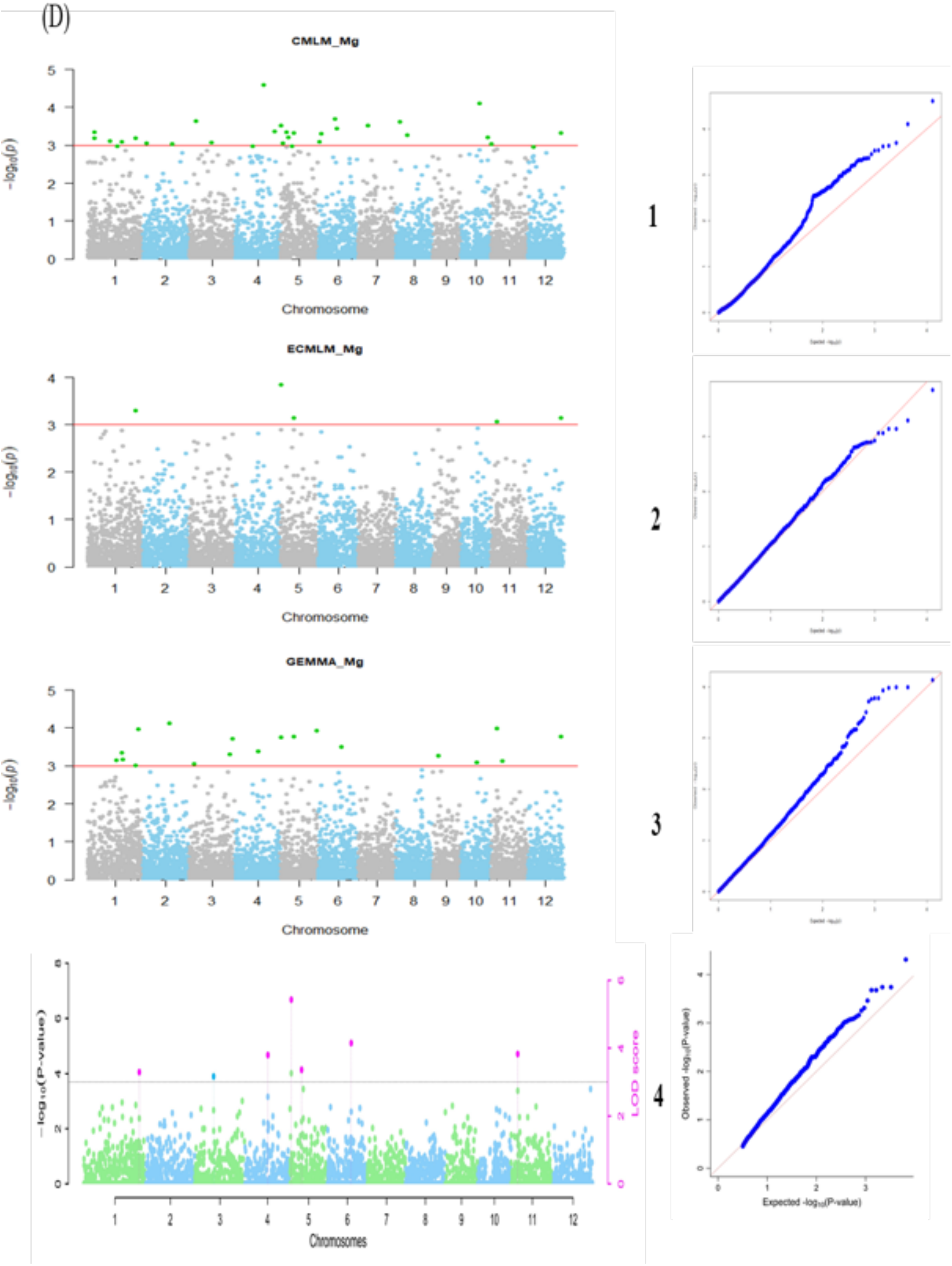

(D)
CMLM_Mg
ECMLM_Mg
GEMMA_Mg
$-\log_{10}(p)$
Chromosome
$-\log_{10}$(P-value)
LOD score
Chromosomes
1
2
3
4
Observed $-\log_{10}$(P-value)
Expected $-\log_{10}$(P-value)

(E)

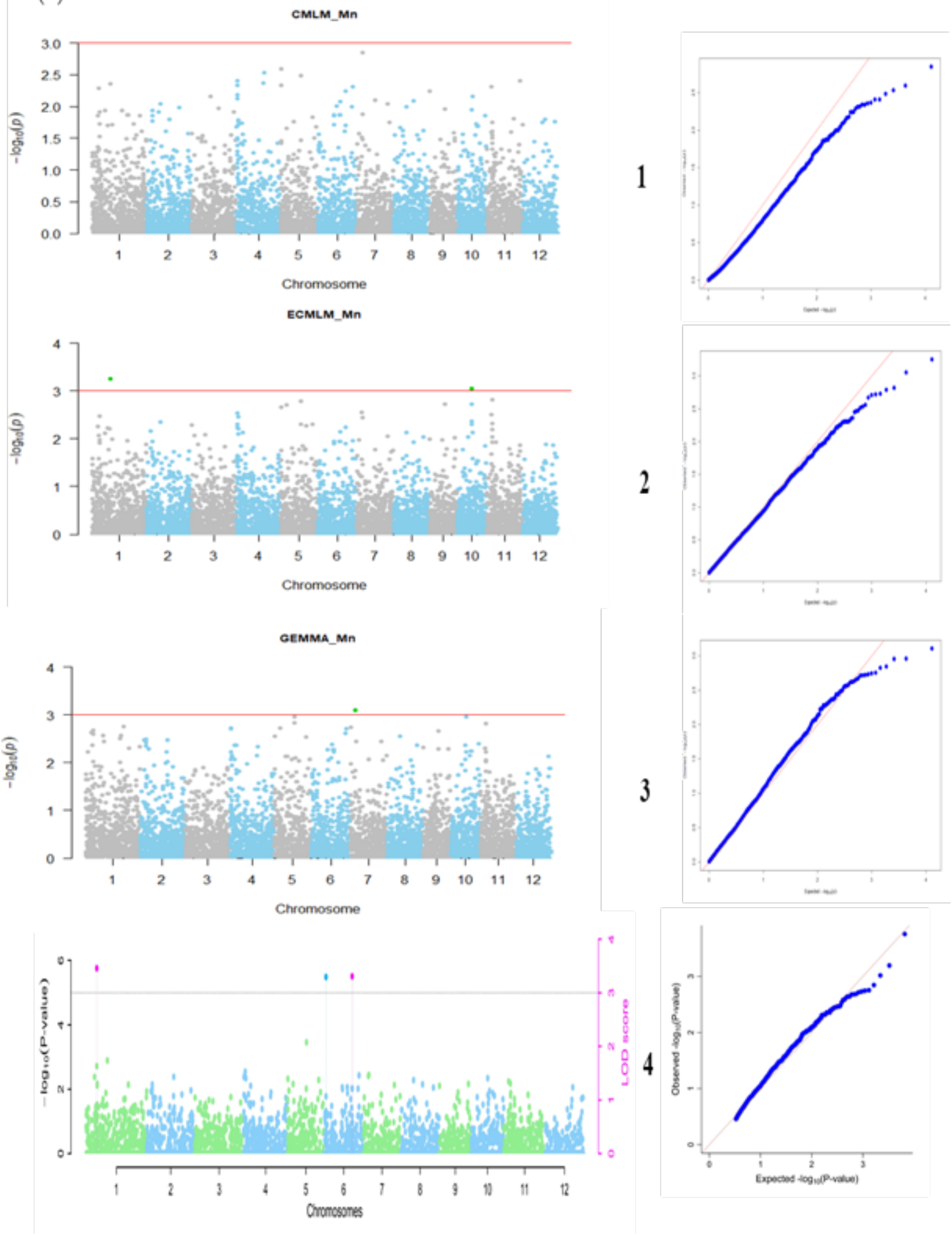
CMLM_Mn
$-\log_{10}(p)$
Chromosome
ECMLM_Mn
$-\log_{10}(p)$
Chromosome
GEMMA_Mn
$-\log_{10}(p)$
Chromosome
$-\log_{10}$(P-value)
LOD score
Chromosomes
Observed $-\log_{10}$(P-value)
Expected $-\log_{10}$(P-value)
1
2
3
4

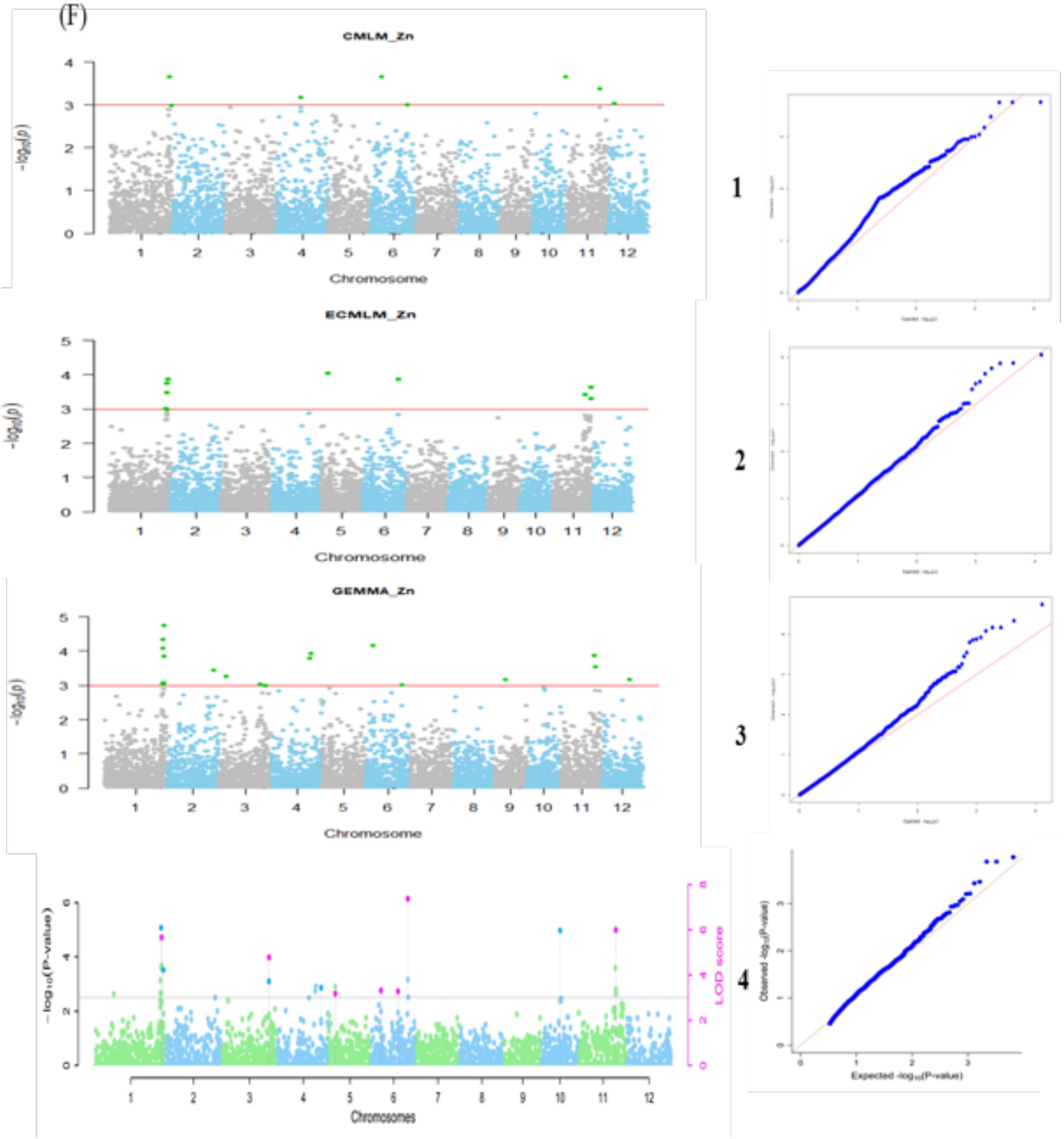

Figure S2. Manhattan plots of GWAS for six minerals. (1). Manhattan plot CMLM model. (2). Manhattan plot for ECMLM model. (3). Manhattan plot for GEMMA model. (4). Manhattan plot for multi-locus models, including mrMLM, FASTmrMLM, FASTmrEMMA, pLARmEB, pKWmEB, ISIS EM-BLASSO. The red horizontal line in the 1, 2, and 3 models is the threshold significant level used in the study to declare a SNP as being significant for measured traits. The green circles above the red line depict the significant SNPs. For 4 model, purple circles above the dashed horizontal line are the significant SNPs identified by any two models of six models, whereas green circles show only those SNPs identified by only one model of six models of multi-locus method. (A) Cu. (B) Fe, (C) K. (D) Mg. (E) Mn. (F) Zn.

1. Cu

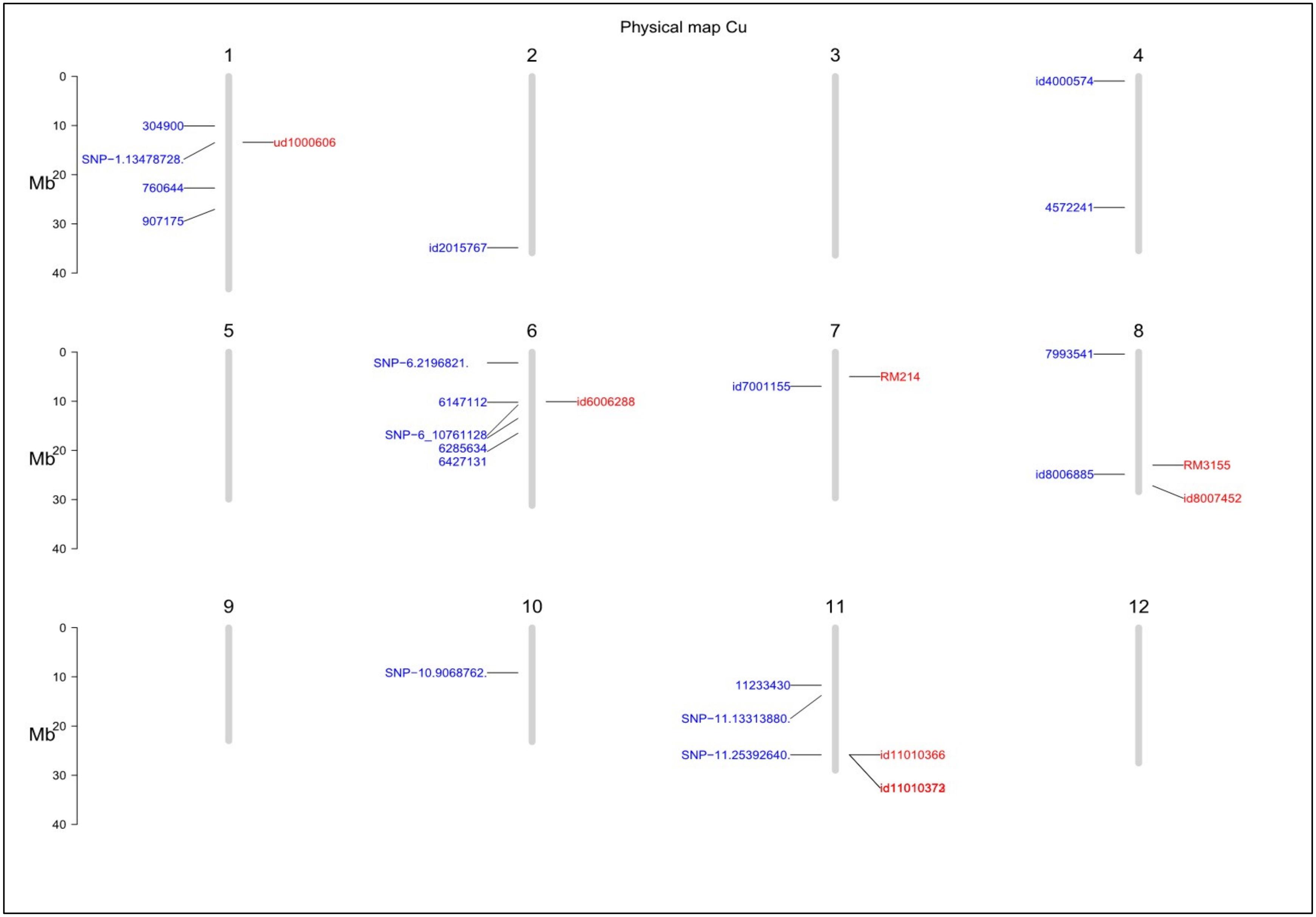

Physical map Cu
1
2
3
4
5
6
7
8
9
10
11
12
Mb
0
10
20
30
40
304900
SNP−1.13478728.
760644
907175
ud1000606
id2015767
id4000574
4572241
SNP−6.2196821.
6147112
SNP−6_10761128
6285634
6427131
id6006288
id7001155
RM214
7993541
id8006885
RM3155
id8007452
SNP−10.9068762.
11233430
SNP−11.13313880.
SNP−11.25392640.
id11010366
id11010372

2. Fe

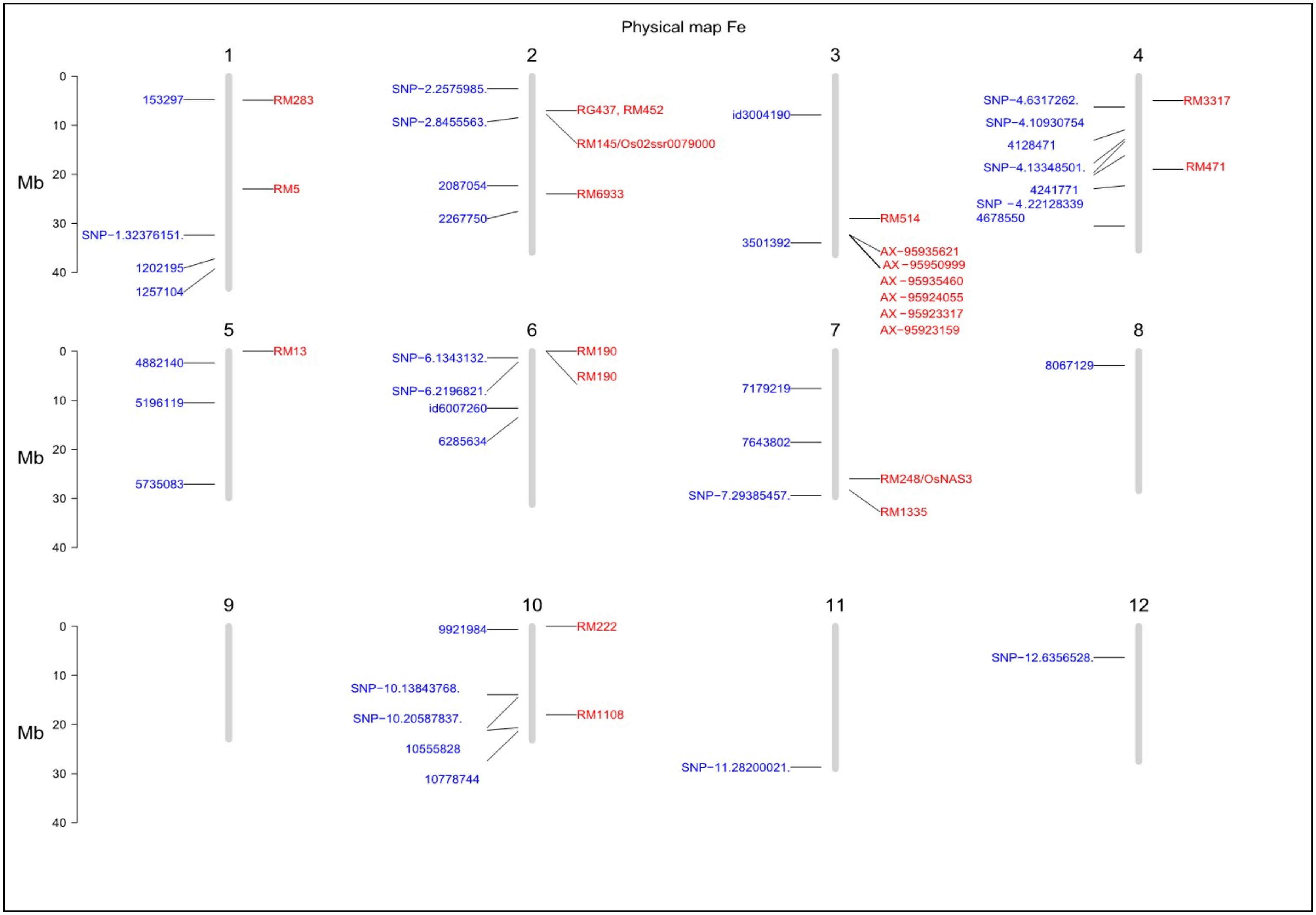

Physical map Fe
1
2
3
4
Mb
0
10
20
30
40
153297
RM283
RM5
SNP−1.32376151.
1202195
1257104
SNP−2.2575985.
SNP−2.8455563.
2087054
2267750
RG437, RM452
RM145/Os02ssr0079000
RM6933
id3004190
3501392
RM514
AX−95935621
AX−95950999
AX−95935460
AX−95924055
AX−95923317
AX−95923159
SNP−4.6317262.
SNP−4.10930754
4128471
SNP−4.13348501.
4241771
SNP −4.22128339
4678550
RM3317
RM471
5
6
7
8
4882140
5196119
5735083
RM13
SNP−6.1343132.
SNP−6.2196821.
id6007260
6285634
RM190
RM190
7179219
7643802
SNP−7.29385457.
RM248/OsNAS3
RM1335
8067129
9
10
11
12
9921984
SNP−10.13843768.
SNP−10.20587837.
10555828
10778744
RM222
RM1108
SNP−11.28200021.
SNP−12.6356528.

3. K

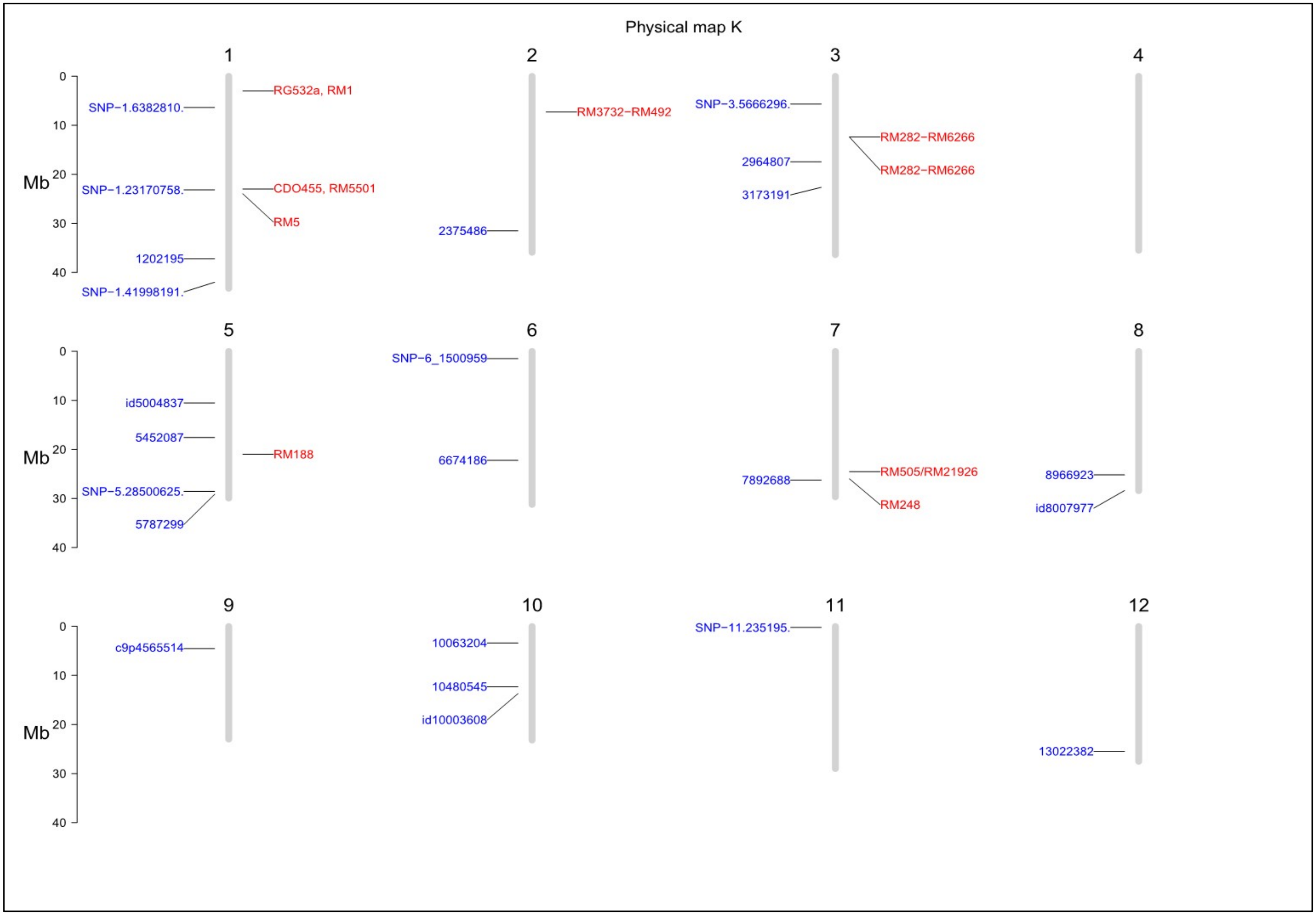
Physical map K
1
2
3
4
5
6
7
8
9
10
11
12
Mb
0
10
20
30
40
SNP−1.6382810.
SNP−1.23170758.
1202195
SNP−1.41998191.
RG532a, RM1
CDO455, RM5501
RM5
RM3732−RM492
2375486
SNP−3.5666296.
2964807
3173191
RM282−RM6266
RM282−RM6266
id5004837
5452087
SNP−5.28500625.
5787299
RM188
SNP−6_1500959
6674186
7892688
RM505/RM21926
RM248
8966923
id8007977
c9p4565514
10063204
10480545
id10003608
SNP−11.235195.
13022382

4. Mg

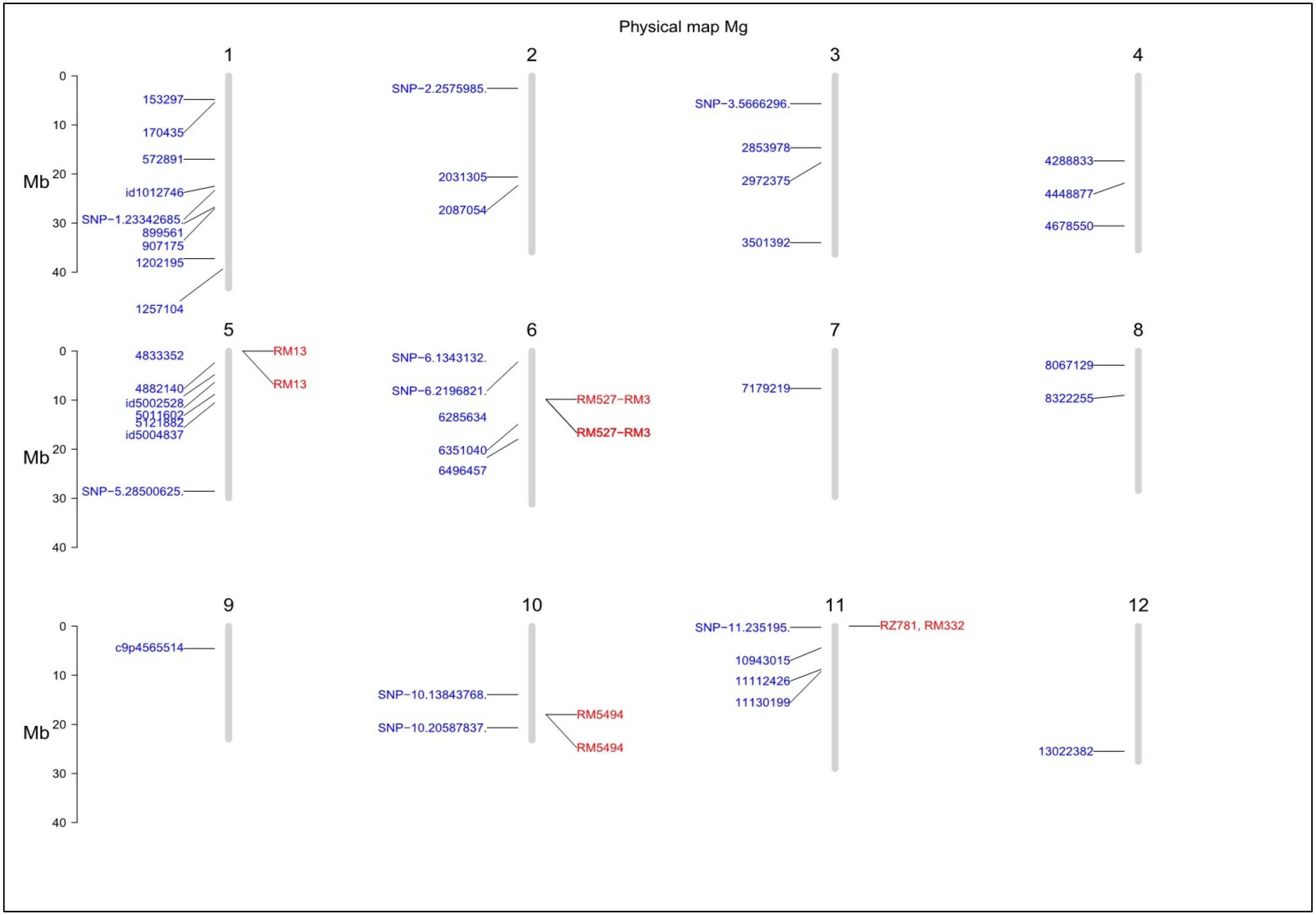

Physical map Mg
1
2
3
4
5
6
7
8
9
10
11
12
Mb
153297
170435
572891
id1012746
SNP−1.23342685.
899561
907175
1202195
1257104
SNP−2.2575985.
2031305
2087054
SNP−3.5666296.
2853978
2972375
3501392
4288833
4448877
4678550
4833352
4882140
id5002528
5011602
5121882
id5004837
SNP−5.28500625.
RM13
RM13
SNP−6.1343132.
SNP−6.2196821.
6285634
6351040
6496457
RM527−RM3
RM527−RM3
7179219
8067129
8322255
c9p4565514
SNP−10.13843768.
SNP−10.20587837.
RM5494
RM5494
SNP−11.235195.
10943015
11112426
11130199
RZ781, RM332
13022382

5. Mn

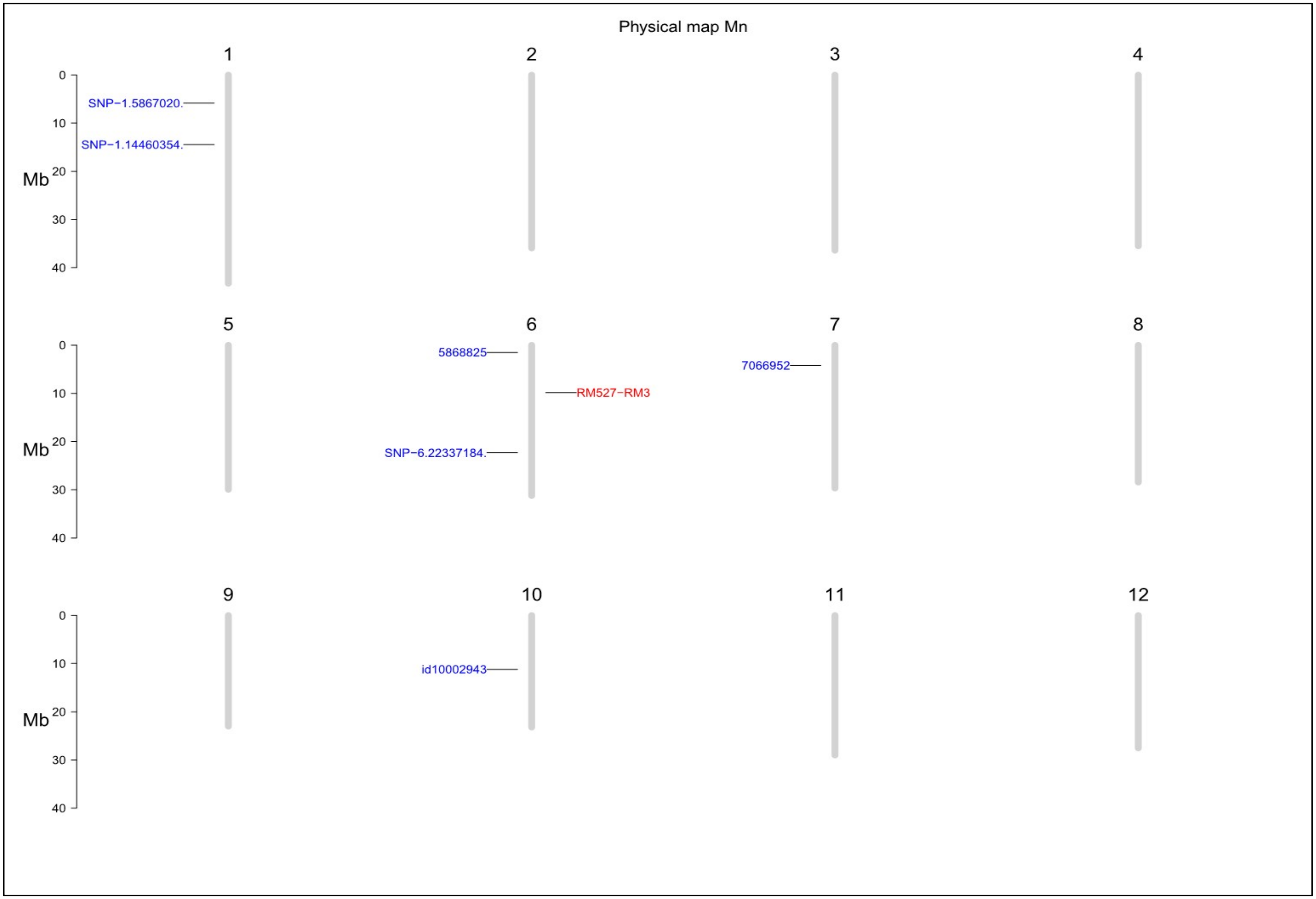

Physical map Mn
1
2
3
4
SNP−1.5867020.
SNP−1.14460354.
Mb
0
10
20
30
40
5
6
7
8
5868825
RM527−RM3
7066952
SNP−6.22337184.
9
10
11
12
id10002943

6. Zn

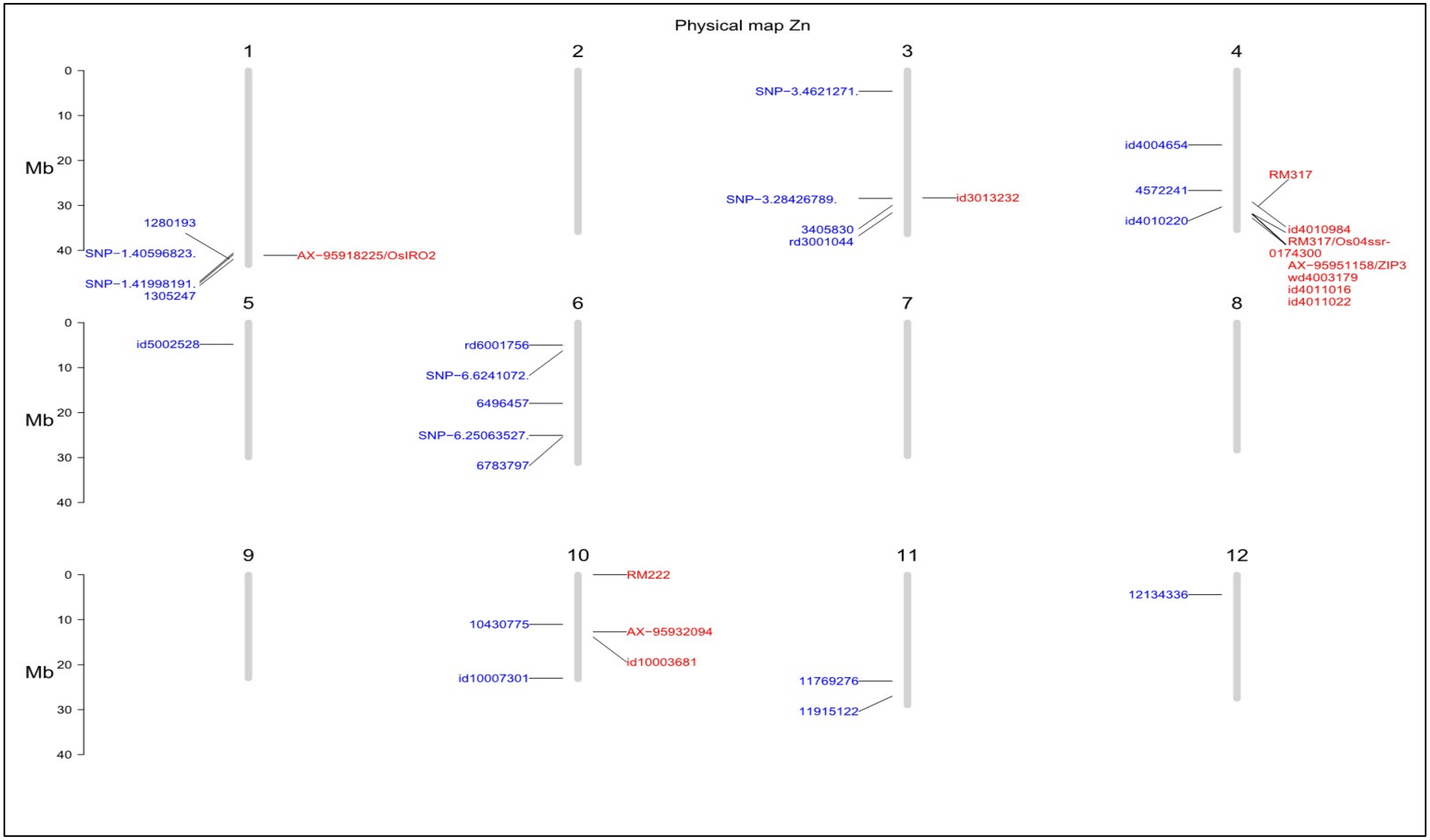

Figure S3. Physical map of the significant SNPs at –log10P ≥ 3.0 significant threshold value for six mineral elements. (1) Cu. (2). Fe (3). K (4) Mg (5) Mn (6) Zn. Vertical lines indicate rice chromosomes. The significant SNPs detected in this study are marked as blue color on the left side of the chromosomes. The previously reported genes/markers are displayed as red color on the right of the chromosomes.

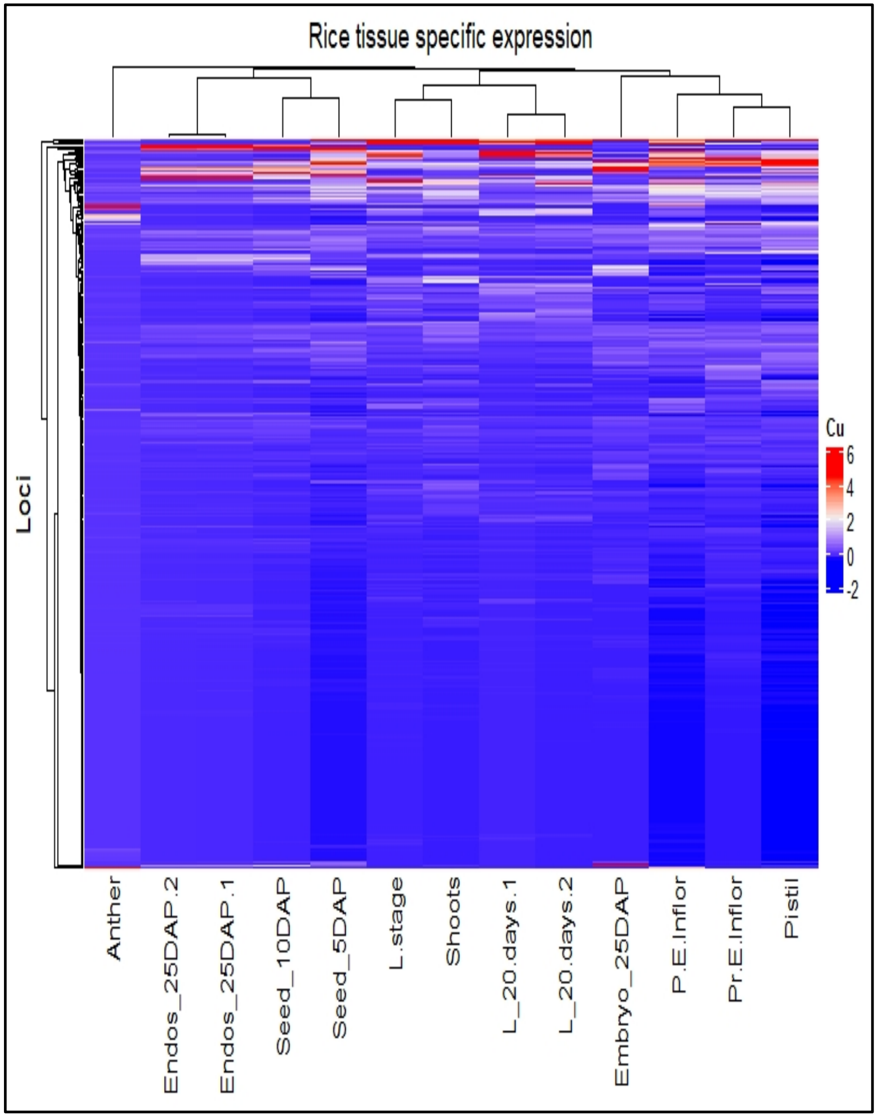
Rice tissue specific expression
Loci
Cu
6
4
2
0
-2
Anther
Endos_25DAP.2
Endos_25DAP.1
Seed_10DAP
Seed_5DAP
L.stage
Shoots
L_20.days.1
L_20.days.2
Embryo_25DAP
P.E.Inflor
Pr.E.Inflor
Pistil
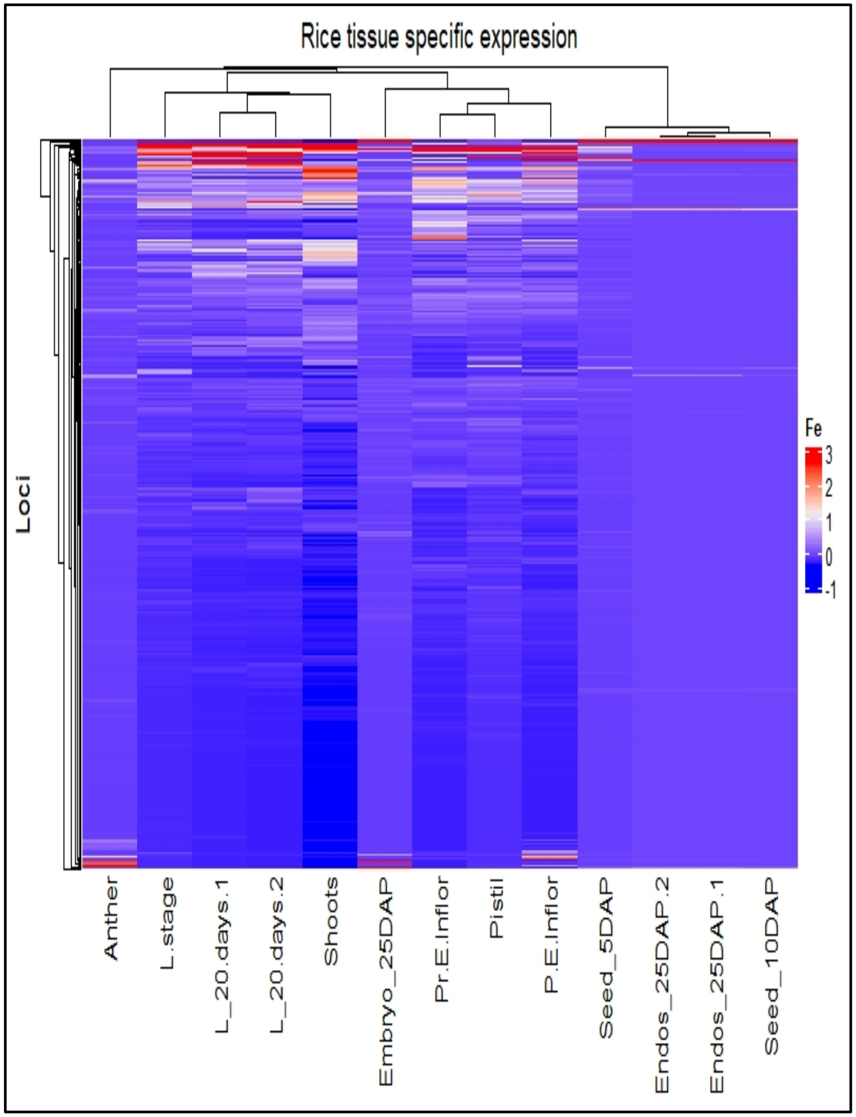
Rice tissue specific expression
Loci
Fe
3
2
1
0
-1
Anther
L.stage
L_20.days.1
L_20.days.2
Shoots
Embryo_25DAP
Pr.E.Inflor
Pistil
P.E.Inflor
Seed_5DAP
Endos_25DAP.2
Endos_25DAP.1
Seed_10DAP

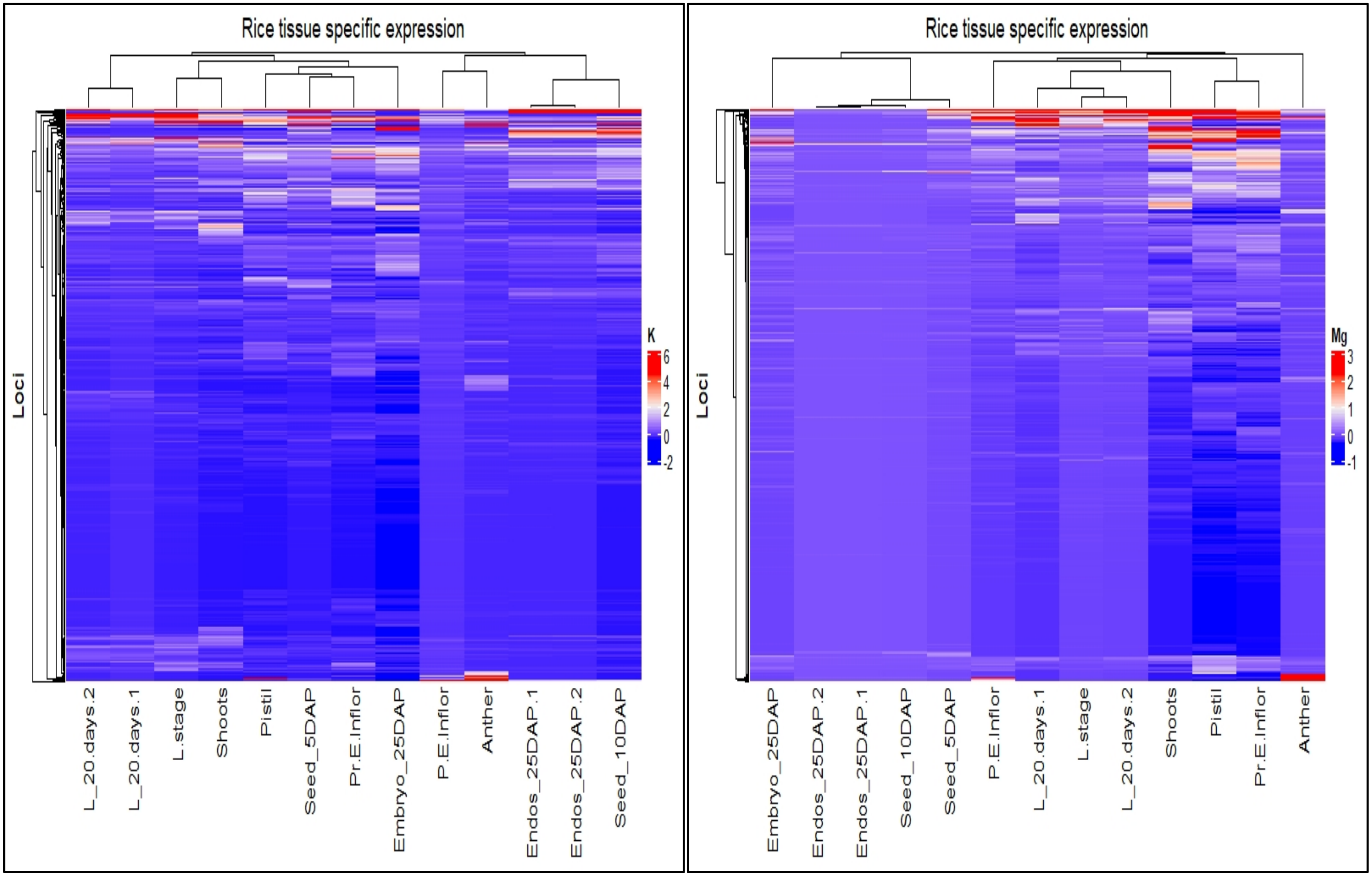
Rice tissue specific expression
Loci
K
6
4
2
0
-2
L_20.days.2
L_20.days.1
L.stage
Shoots
Pistil
Seed_5DAP
Pr.E.Inflor
Embryo_25DAP
P.E.Inflor
Anther
Endos_25DAP.1
Endos_25DAP.2
Seed_10DAP
Rice tissue specific expression
Loci
Mg
3
2
1
0
-1
Embryo_25DAP
Endos_25DAP.2
Endos_25DAP.1
Seed_10DAP
Seed_5DAP
P.E.Inflor
L_20.days.1
L.stage
L_20.days.2
Shoots
Pistil
Pr.E.Inflor
Anther

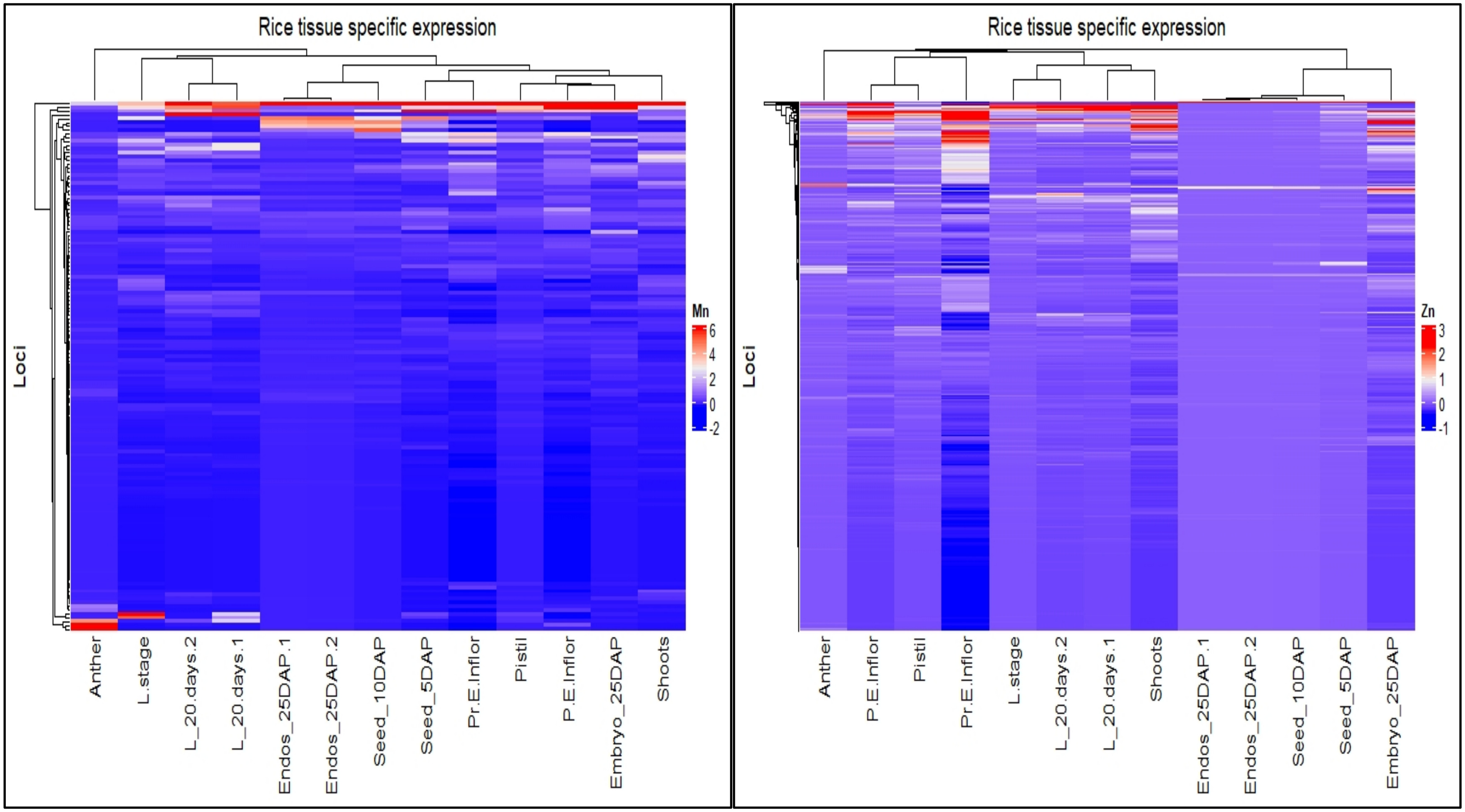

Figure S4. Heatmap of In-silico gene expression analysis results for the six mineral elements of the study in Nipponbare. Description of abbreviated terms: L.stage = Leaf stage; L_20.days.1 = Leaves-20 days; L_20.days.2 = Leaves-20 days; Endos_25DAP.1 = Endosperm- 25 DAP; Endos_25DAP.2 = Endosperm- 25 DAP; Seed_10DAP = Seed after 10 days of pollination; Seed_5DAP = Seed after 5 days of pollination; Pr.E.Inflor = Pre-emergence inflorescence; P.E.Inflor = Post-emergence inflorescence; Embryo_25DAP = Seed embryo after 25 days of pollination.

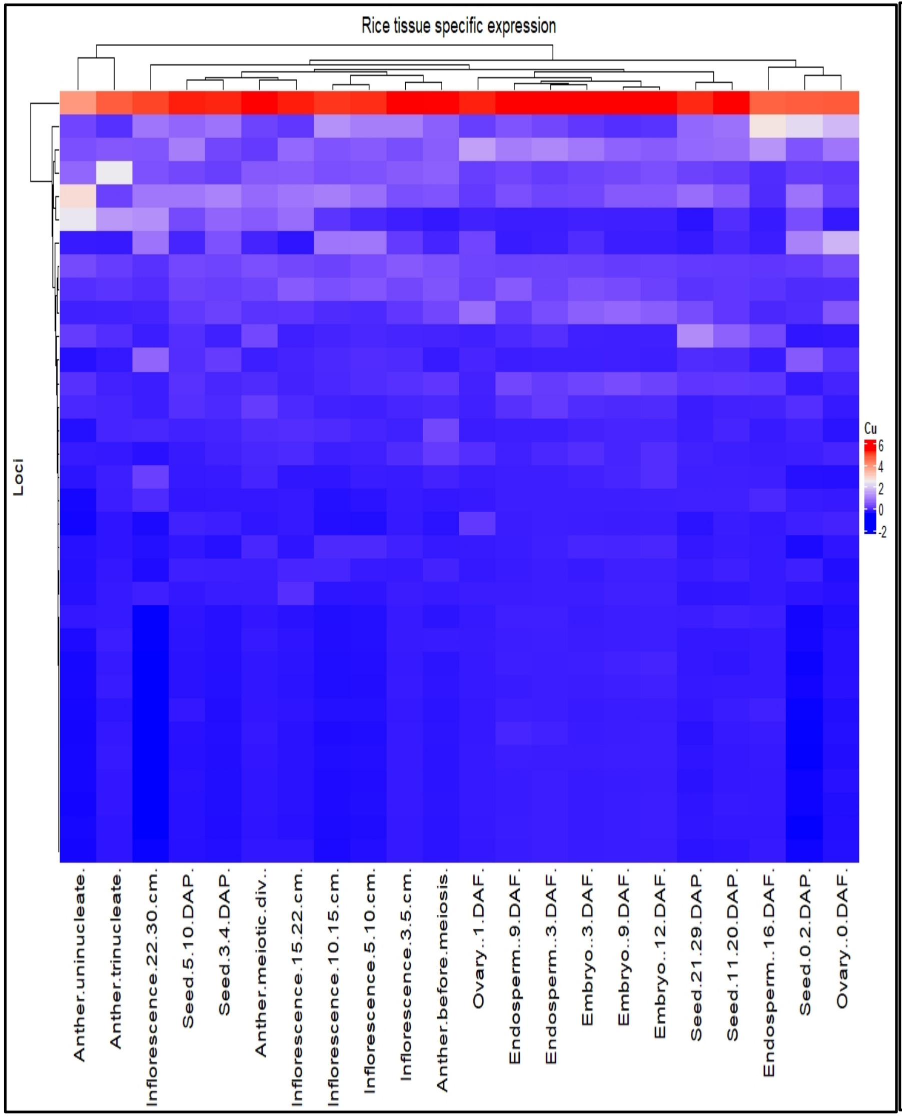

Rice tissue specific expression
Loci
Cu
6
4
2
0
-2
Anther.uninucleate.
Anther.trinucleate.
Inflorescence.22.30.cm.
Seed.5.10.DAP.
Seed.3.4.DAP.
Anther.meiotic.div..
Inflorescence.15.22.cm.
Inflorescence.10.15.cm.
Inflorescence.5.10.cm.
Inflorescence.3.5.cm.
Anther.before.meiosis.
Ovary..1.DAF.
Endosperm..9.DAF.
Endosperm..3.DAF.
Embryo..3.DAF.
Embryo..9.DAF.
Embryo..12.DAF.
Seed.21.29.DAP.
Seed.11.20.DAP.
Endosperm..16.DAF.
Seed.0.2.DAP.
Ovary..0.DAF.

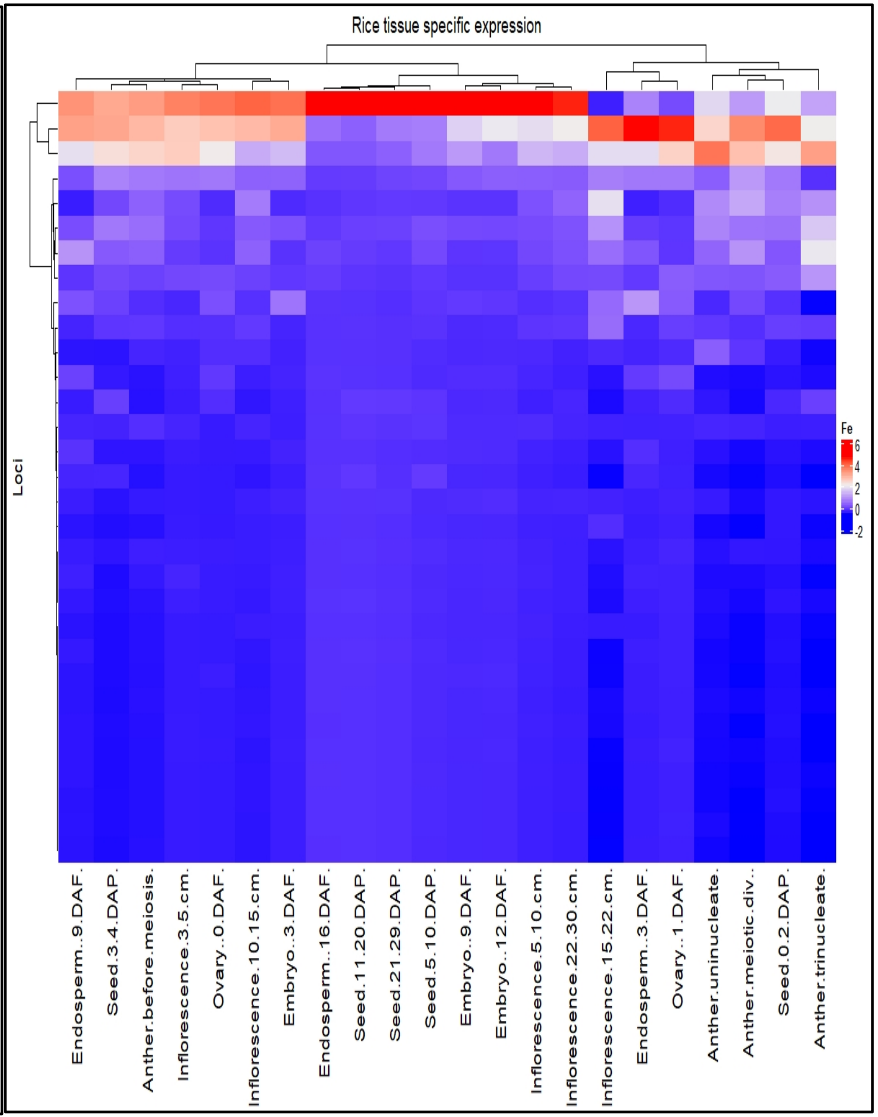

Rice tissue specific expression
Loci
Fe
6
4
2
0
-2
Endosperm..9.DAF.
Seed.3.4.DAP.
Anther.before.meiosis.
Inflorescence.3.5.cm.
Ovary..0.DAF.
Inflorescence.10.15.cm.
Embryo..3.DAF.
Endosperm..16.DAF.
Seed.11.20.DAP.
Seed.21.29.DAP.
Seed.5.10.DAP.
Embryo..9.DAF.
Embryo..12.DAF.
Inflorescence.5.10.cm.
Inflorescence.22.30.cm.
Inflorescence.15.22.cm.
Endosperm..3.DAF.
Ovary..1.DAF.
Anther.uninucleate.
Anther.meiotic.div..
Seed.0.2.DAP.
Anther.trinucleate.

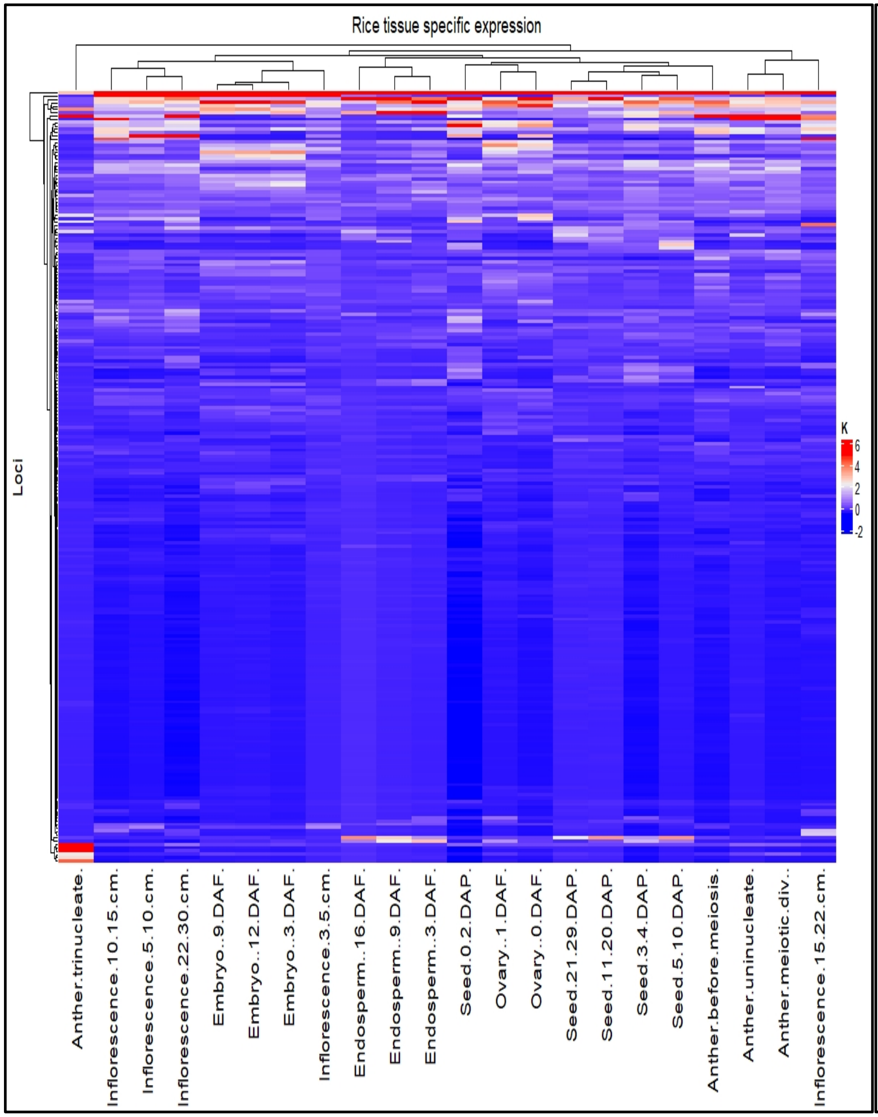
Rice tissue specific expression
Loci
K
6
4
2
0
-2
Anther.trinucleate.
Inflorescence.10.15.cm.
Inflorescence.5.10.cm.
Inflorescence.22.30.cm.
Embryo..9.DAF.
Embryo..12.DAF.
Embryo..3.DAF.
Inflorescence.3.5.cm.
Endosperm..16.DAF.
Endosperm..9.DAF.
Endosperm..3.DAF.
Seed.0.2.DAP.
Ovary..1.DAF.
Ovary..0.DAF.
Seed.21.29.DAP.
Seed.11.20.DAP.
Seed.3.4.DAP.
Seed.5.10.DAP.
Anther.before.meiosis.
Anther.uninucleate.
Anther.meiotic.div..
Inflorescence.15.22.cm.

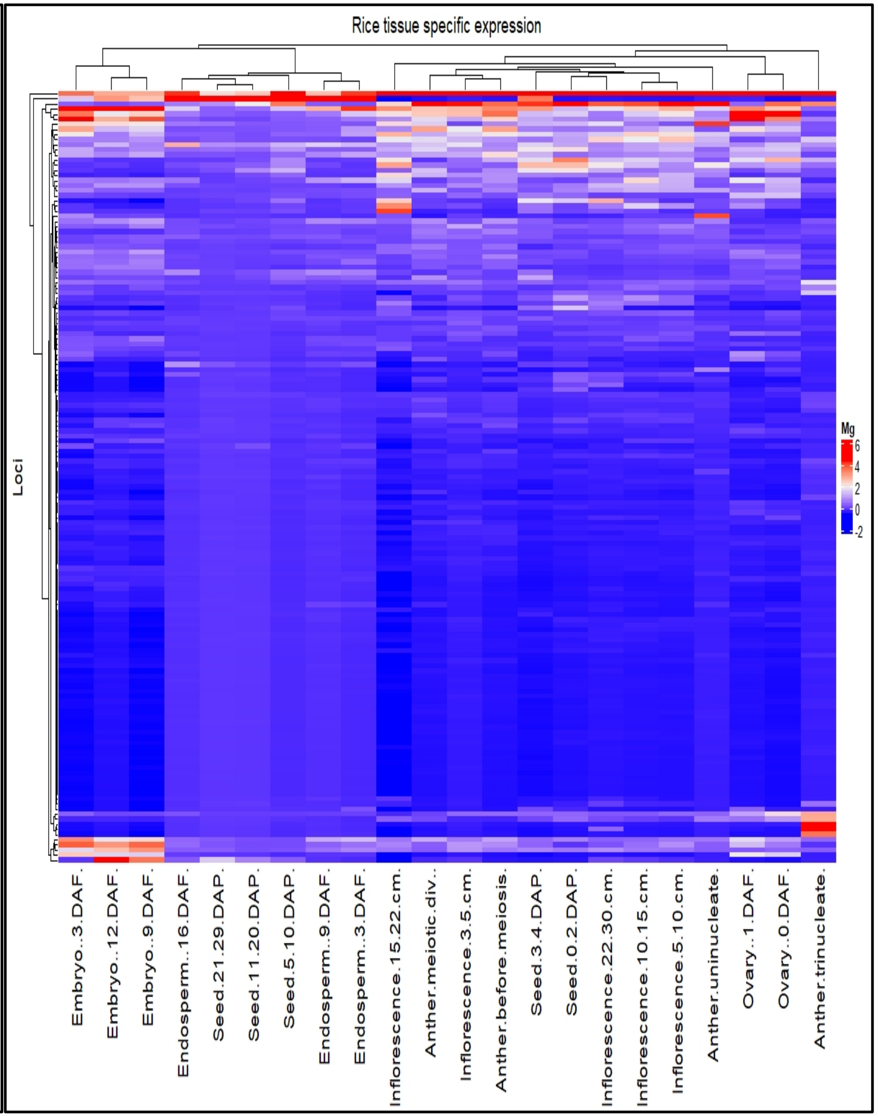
Rice tissue specific expression
Loci
Mg
6
4
2
0
-2
Embryo..3.DAF.
Embryo..12.DAF.
Embryo..9.DAF.
Endosperm..16.DAF.
Seed.21.29.DAP.
Seed.11.20.DAP.
Seed.5.10.DAP.
Endosperm..9.DAF.
Endosperm..3.DAF.
Inflorescence.15.22.cm.
Anther.meiotic.div..
Inflorescence.3.5.cm.
Anther.before.meiosis.
Seed.3.4.DAP.
Seed.0.2.DAP.
Inflorescence.22.30.cm.
Inflorescence.10.15.cm.
Inflorescence.5.10.cm.
Anther.uninucleate.
Ovary..1.DAF.
Ovary..0.DAF.
Anther.trinucleate.

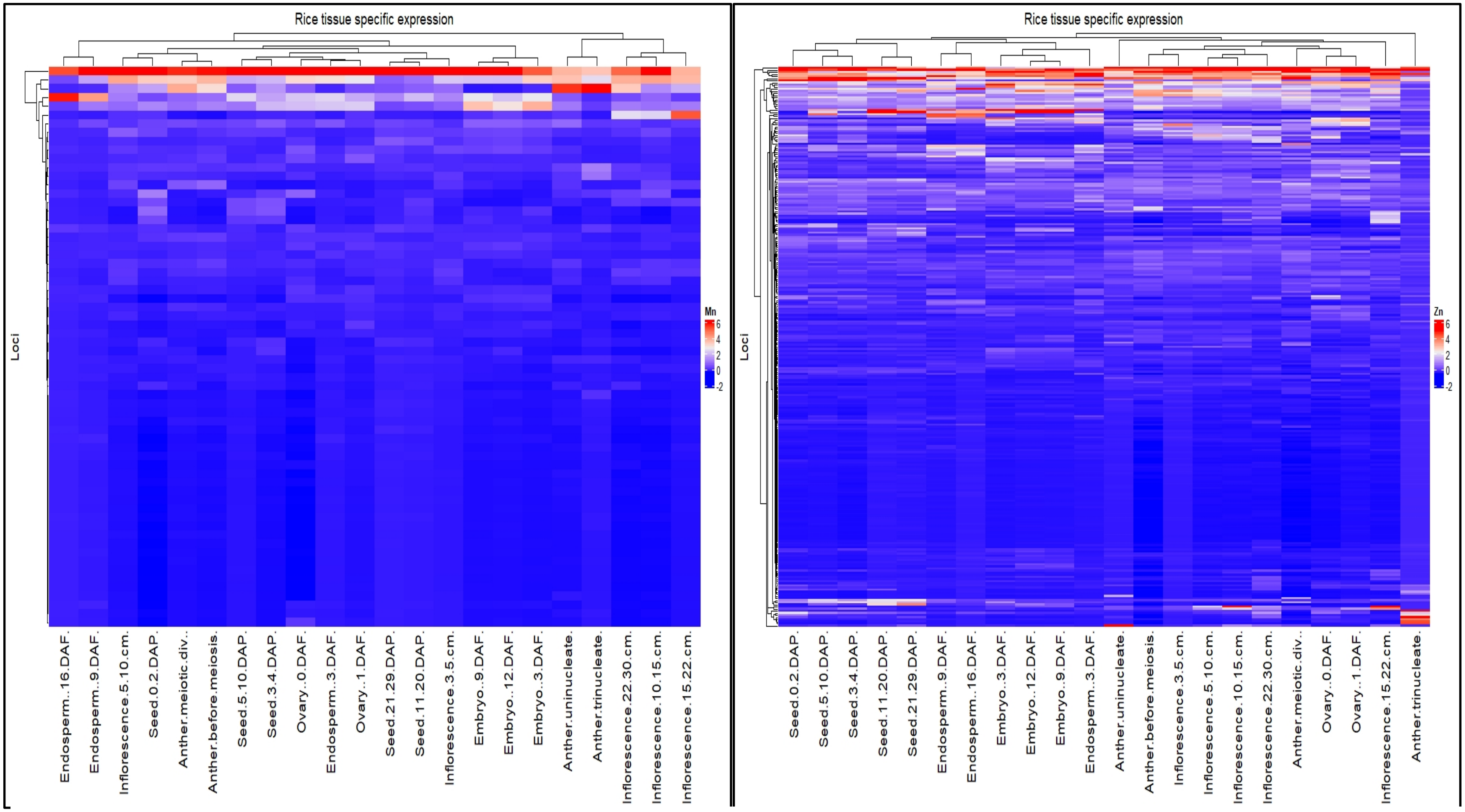

Figure S5. Heatmap of In-silico gene expression analysis results for the six mineral elements of the study in IR64. Description of abbreviated terms: Endosperm..16.DAF = Endosperm (16 DAF); Endosperm..9.DAF = Endosperm (9 DAF); Inflorescence.5.10.cm = Inflorescence(5-10 cm); Seed.0.2.DAP = Seed(0-2 DAP); Anther.meiotic.div. = Anther(meiotic division); Anther.before.meiosis = Anther(before meiosis); Seed.5.10.DAP = Seed(5-10 DAP); Seed.3.4.DAP = Seed(3-4 DAP); Ovary..0.DAF = Ovary (0 DAF); Endosperm..3.DAF = Endosperm (3 DAF); Ovary..1.DAF = Ovary (1 DAF); Seed.21.29.DAP = Seed(21-29 DAP); Seed.11.20.DAP = Seed(11-20 DAP); Inflorescence.3.5.cm = Inflorescence(3-5 cm); Embryo..9.DAF = Embryo (9 DAF); Embryo..12.DAF = Embryo (12 DAF); Embryo..3.DAF = Embryo (3 DAF); Anther.uninucleate = Anther(uninucleate); Anther.trinucleate = Anther(trinucleate); Inflorescence.22.30.cm= Inflorescence(22-30 cm); Inflorescence.10.15.cm = Inflorescence(10-15 cm); Inflorescence.15.22.cm = Inflorescence(15-22 cm).

1. Cu

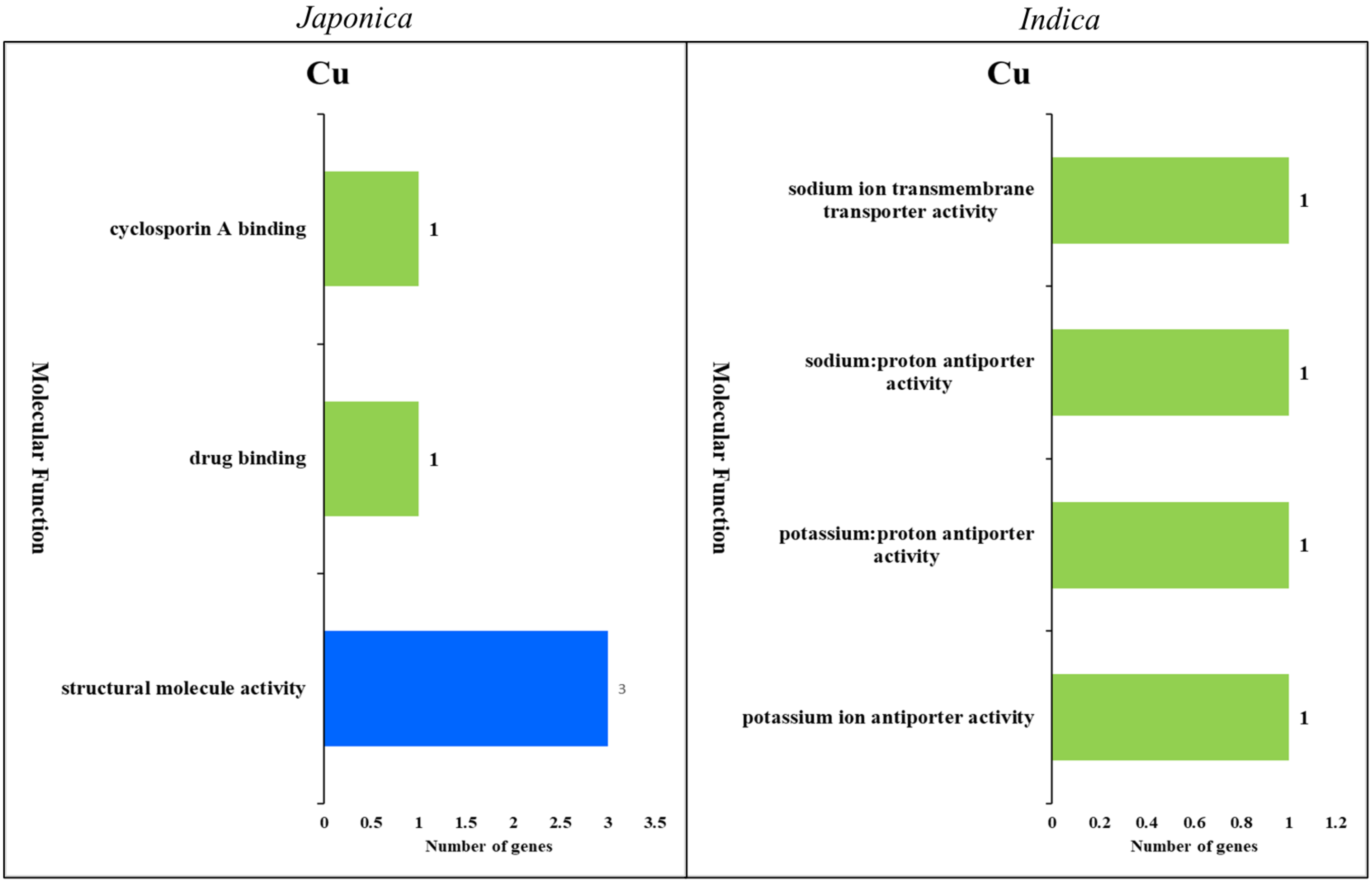
Japonica
Indica
Cu
Molecular Function
cyclosporin A binding
drug binding
structural molecule activity
1
1
3
0 0.5 1 1.5 2 2.5 3 3.5
Number of genes
Cu
Molecular Function
sodium ion transmembrane transporter activity
sodium:proton antiporter activity
potassium:proton antiporter activity
potassium ion antiporter activity
1
1
1
1
0 0.2 0.4 0.6 0.8 1 1.2
Number of genes

2. Fe

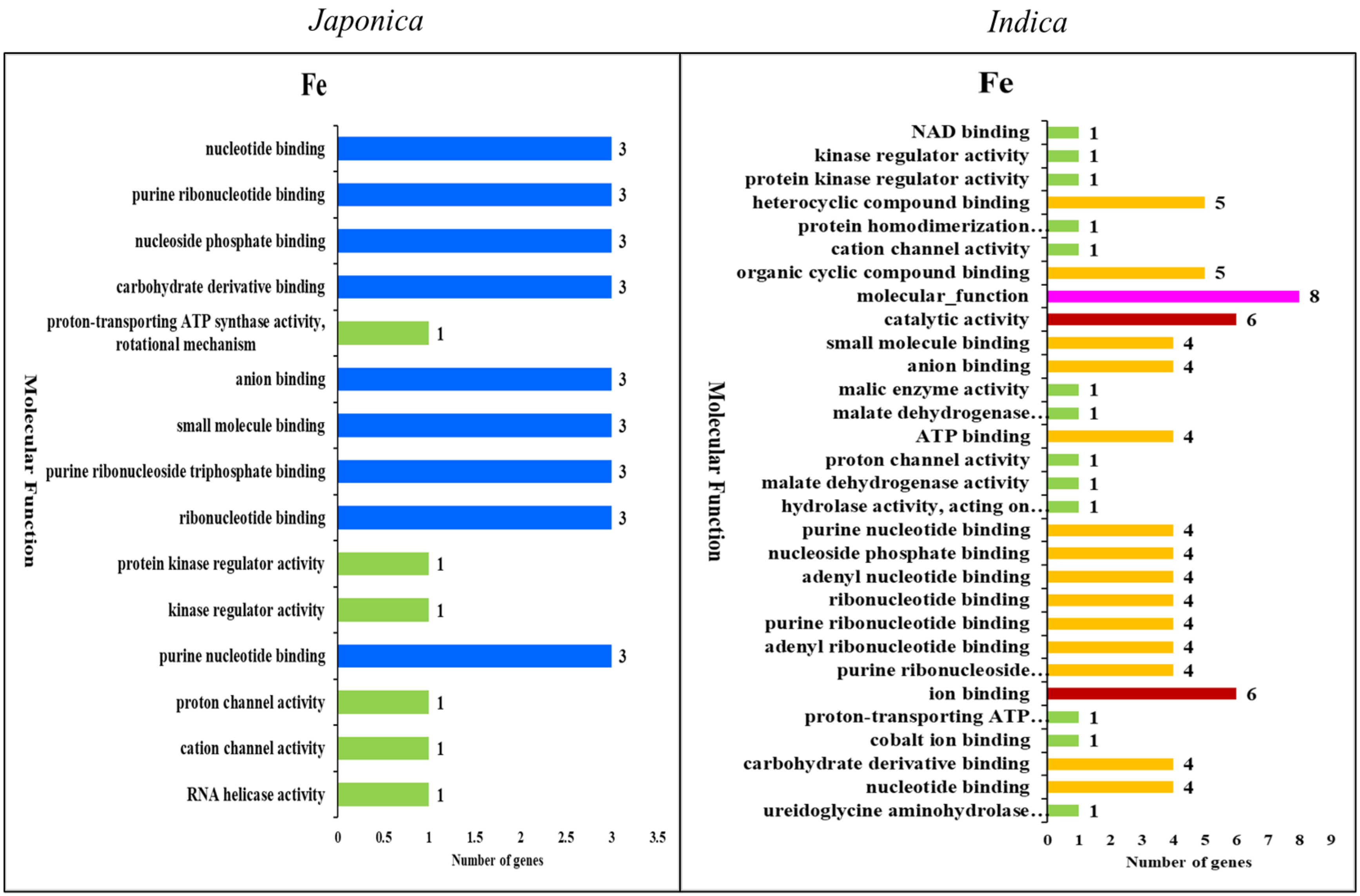
Japonica
Indica
Fe
nucleotide binding
purine ribonucleotide binding
nucleoside phosphate binding
carbohydrate derivative binding
proton-transporting ATP synthase activity, rotational mechanism
anion binding
small molecule binding
purine ribonucleoside triphosphate binding
ribonucleotide binding
protein kinase regulator activity
kinase regulator activity
purine nucleotide binding
proton channel activity
cation channel activity
RNA helicase activity
3
1
0
0.5
1.5
2
2.5
3.5
Number of genes
Molecular Function
Fe
NAD binding
kinase regulator activity
protein kinase regulator activity
heterocyclic compound binding
protein homodimerization…
cation channel activity
organic cyclic compound binding
molecular_function
catalytic activity
small molecule binding
anion binding
malic enzyme activity
malate dehydrogenase…
ATP binding
proton channel activity
malate dehydrogenase activity
hydrolase activity, acting on…
purine nucleotide binding
nucleoside phosphate binding
adenyl nucleotide binding
ribonucleotide binding
purine ribonucleotide binding
adenyl ribonucleotide binding
purine ribonucleoside…
ion binding
proton-transporting ATP…
cobalt ion binding
carbohydrate derivative binding
nucleotide binding
ureidoglycine aminohydrolase…
0 1 2 3 4 5 6 7 8 9
Number of genes
Molecular Function

3. K

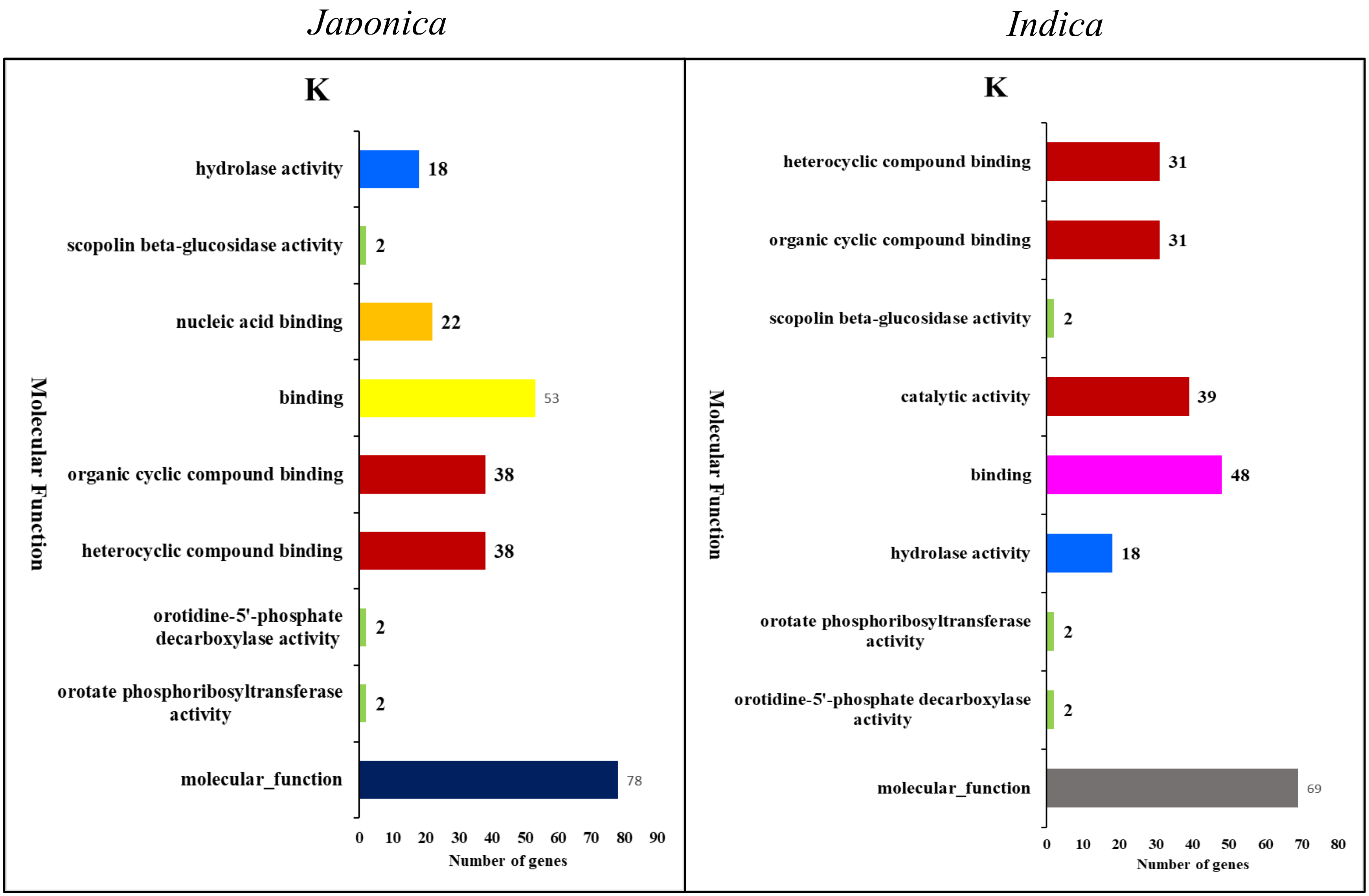
Japonica
Indica
K
hydrolase activity
18
scopolin beta-glucosidase activity
2
nucleic acid binding
22
binding
53
organic cyclic compound binding
38
heterocyclic compound binding
38
orotidine-5'-phosphate decarboxylase activity
2
orotate phosphoribosyltransferase activity
2
molecular_function
78
Molecular Function
0 10 20 30 40 50 60 70 80 90
Number of genes
K
heterocyclic compound binding
31
organic cyclic compound binding
31
scopolin beta-glucosidase activity
2
catalytic activity
39
binding
48
hydrolase activity
18
orotate phosphoribosyltransferase activity
2
orotidine-5'-phosphate decarboxylase activity
2
molecular_function
69
Molecular Function
0 10 20 30 40 50 60 70 80
Number of genes

4. Mg

Indica

Japonica

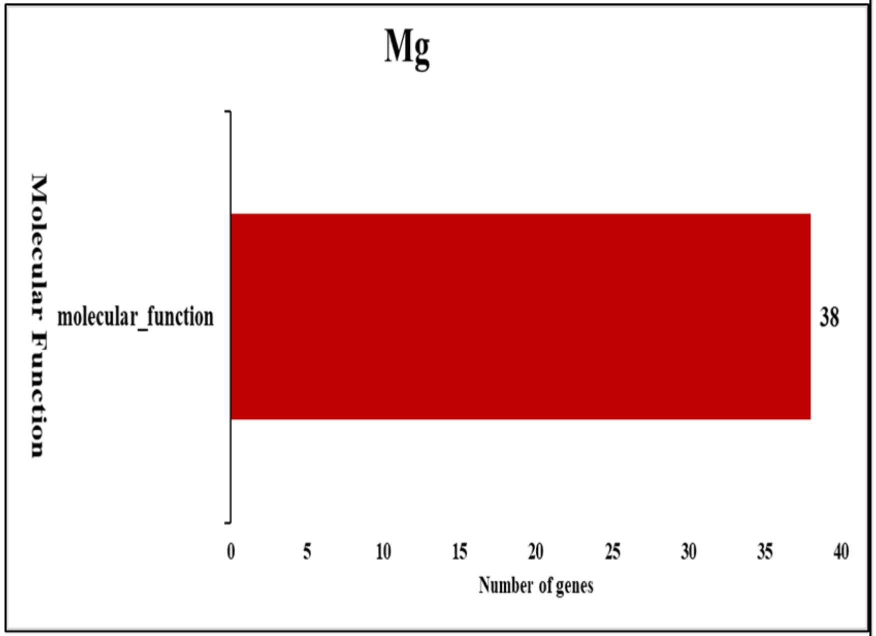

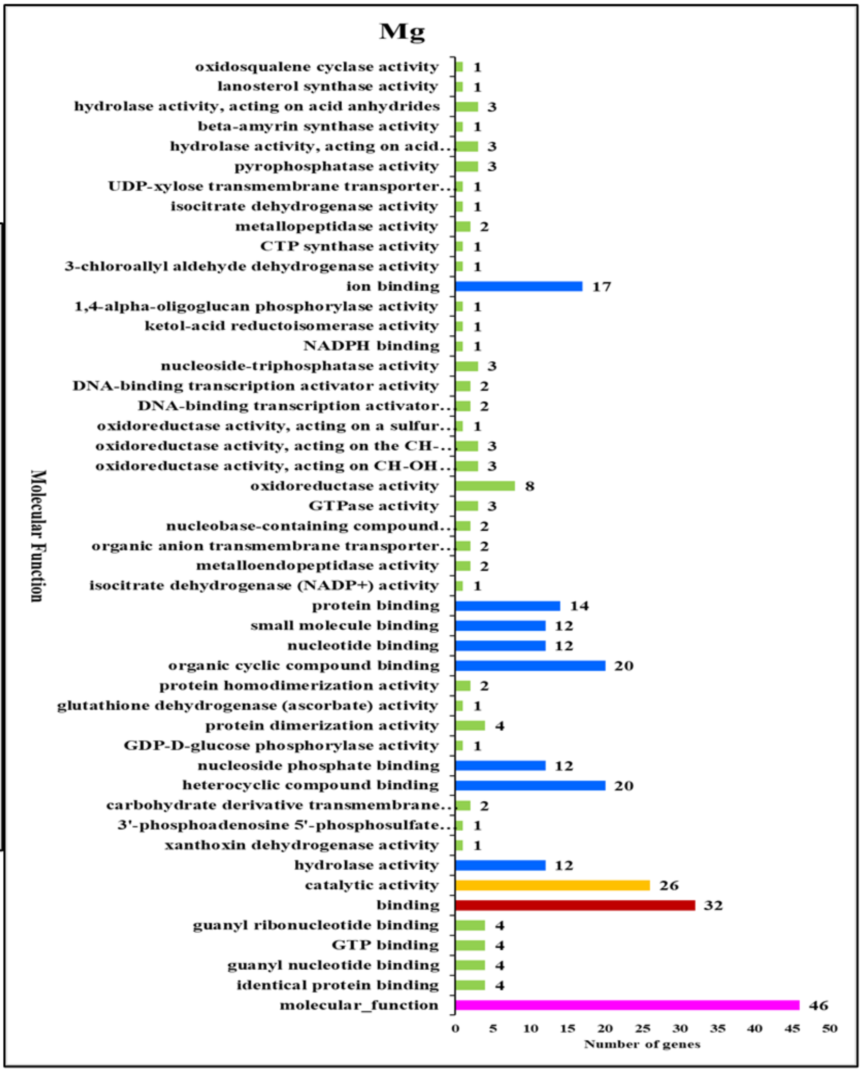

5. Mn

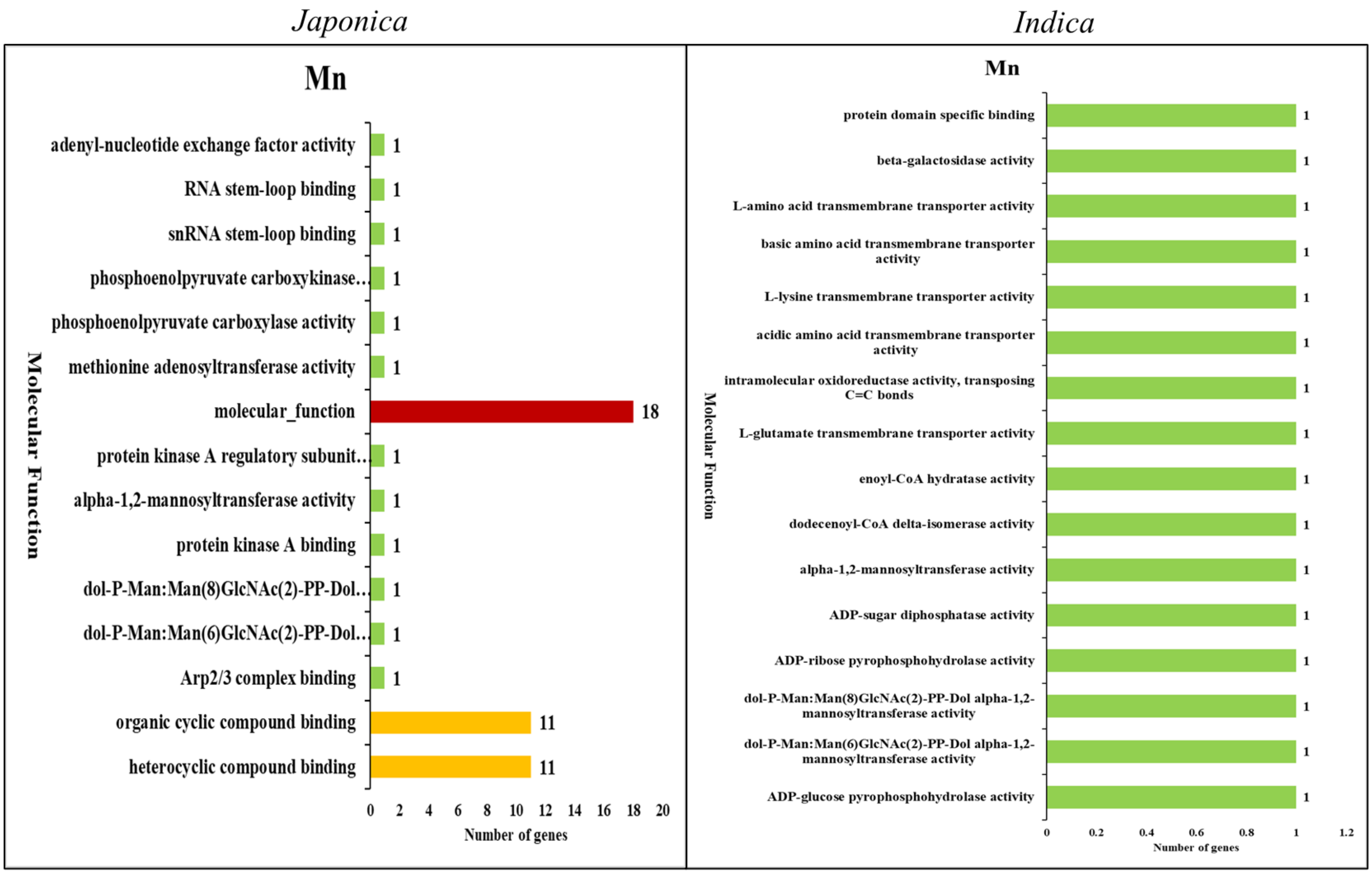
Japonica
Indica
Mn
adenyl-nucleotide exchange factor activity
RNA stem-loop binding
snRNA stem-loop binding
phosphoenolpyruvate carboxykinase…
phosphoenolpyruvate carboxylase activity
methionine adenosyltransferase activity
molecular_function
protein kinase A regulatory subunit…
alpha-1,2-mannosyltransferase activity
protein kinase A binding
dol-P-Man:Man(8)GlcNAc(2)-PP-Dol…
dol-P-Man:Man(6)GlcNAc(2)-PP-Dol…
Arp2/3 complex binding
organic cyclic compound binding
heterocyclic compound binding
Molecular Function
Number of genes
0 2 4 6 8 10 12 14 16 18 20
Mn
protein domain specific binding
beta-galactosidase activity
L-amino acid transmembrane transporter activity
basic amino acid transmembrane transporter activity
L-lysine transmembrane transporter activity
acidic amino acid transmembrane transporter activity
intramolecular oxidoreductase activity, transposing C=C bonds
L-glutamate transmembrane transporter activity
enoyl-CoA hydratase activity
dodecenoyl-CoA delta-isomerase activity
alpha-1,2-mannosyltransferase activity
ADP-sugar diphosphatase activity
ADP-ribose pyrophosphohydrolase activity
dol-P-Man:Man(8)GlcNAc(2)-PP-Dol alpha-1,2-mannosyltransferase activity
dol-P-Man:Man(6)GlcNAc(2)-PP-Dol alpha-1,2-mannosyltransferase activity
ADP-glucose pyrophosphohydrolase activity
Molecular Function
Number of genes
0 0.2 0.4 0.6 0.8 1 1.2

6. Zn

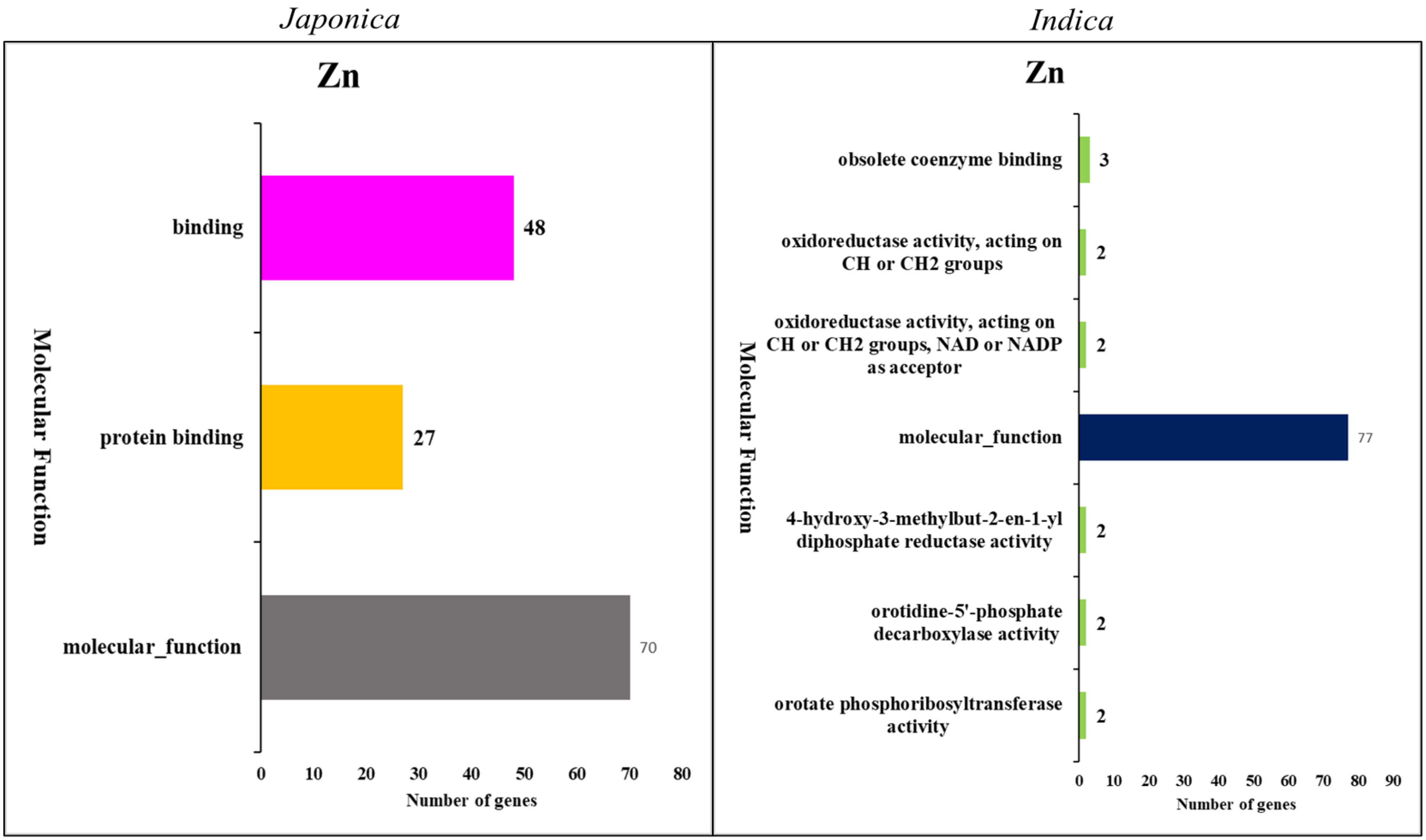

Figure S6. Gene enrichment analysis result, depicting potential molecular functional GO terms for 1.) Cu, 2.) Fe, 3.) K, 4.) Mg, 5.) Mn and 6.) Zn.
